# Stabilization of Monomeric Tau Protein by All D-Enantiomeric Peptide Ligands as Therapeutic Strategy for Alzheimer’s Disease and Other Tauopathies

**DOI:** 10.3390/ijms24032161

**Published:** 2023-01-21

**Authors:** Tim Altendorf, Ian Gering, Beatrix Santiago-Schübel, Selma Aghabashlou Saisan, Gültekin Tamgüney, Markus Tusche, Dominik Honold, Sarah Schemmert, Wolfgang Hoyer, Jeannine Mohrlüder, Dieter Willbold

**Affiliations:** 1 Institut für Biologische Informationsprozesse, IBI-7, Forschungszentrum Jülich, 52425 Jülich, Germany; 2 Institut für Physikalische Biologie, Heinrich-Heine-Universität Düsseldorf, 40225 Düsseldorf, Germany; 3 Zentralinstitut für Engineering, Elektronik und Analytik, ZEA-3, Forschungszentrum Jülich, 52425 Jülich, Germany

**Keywords:** tauopathies, Alzheimer’s disease, all D-enantiomeric peptides, mirror-image phage display, tau aggregation

## Abstract

Alzheimer’s disease and other tauopathies are the world’s leading causes of dementia and memory loss. These diseases are thought to be caused by the misfolding and aggregation of the intracellular tau protein, ultimately leading to neurodegeneration. The tau protein is involved in a multitude of different neurodegenerative diseases. During the onset of tauopathies, tau undergoes structural changes and posttranslational modifications and aggregates into amyloid fibrils that are able to spread with a prion-like behavior. Up to now, there is no therapeutic agent which effectively controls or reverses the disease. Most of the therapeutics that were developed and underwent clinical trials targeted misfolded or aggregated forms of tau. In the current manuscript, we present the selection and characterization of two all D-enantiomeric peptides that bind monomeric tau protein with a low nanomolar K_D_, stabilize tau in its monomeric intrinsically disordered conformation, and stop the conversion of monomers into aggregates. We show that the effect of the two all D-enantiomeric peptides is strong enough to stop ongoing tau aggregation in vitro and is able to significantly reduce tau fibril assembly in cell culture. Both compounds may serve as new lead components for the development of therapeutic agents against Alzheimer’s disease and other tauopathies.

## 1. Introduction

Fibrillary deposits of amyloid beta (Aβ) and tau are characteristic for the pathology of Alzheimer’s disease [1]. The aggregation of both proteins is thought to be responsible for the neurodegeneration that occurs during the course of Alzheimer’s disease. In its nontoxic monomeric form, tau serves important functions in neurons [2]. Tau knockout mice show muscle weakness, impairment during fear conditioning, and hyperactivity in a novel environment [3]. The physiological function of tau is tied to microtubules as tau is a microtubule-associated protein (MAPT). Tau was shown to be an intrinsic component for the building, stabilization, maintenance, and degradation of microtubules [4]. These neuroprotective features are lost upon the aggregation of tau. The exact initial cause for aggregation is still not clear. During aggregation, the monomeric protein undergoes a structural shift and together with other monomers forms oligomers and later fibrils. These fibrils are then found as deposits in the brain and are most prevalent in Alzheimer patients [5,6]. The neurodegeneration present in Alzheimer’s disease is linked to a toxic gain of function of the oligomers and fibrils, in addition to the toxic loss of function due to monomer depletion [7]. Many therapeutic approaches were developed to target Aβ pathology but did not yield positive results in clinical trials [8]. Recent studies show that cognitive defects correlate more with tau propagation and hyperphosphorylation [9,10], while Aβ is claimed to be responsible for tau hyperphosphorylation via the GSK3 cascade [11]. Tau hyperphosphorylation is shown to induce the detachment of tau from microtubules and is assumed to precede tau aggregation. This indicates that tau toxicity may play a more active role in neurodegeneration and explains why many tested Aβ-targeting therapeutics fail to deliver their intended effect during clinical trials. Therefore, the tau protein is thought to be an appropriate target for the development of new therapeutic agents. Ideally, new therapeutics should stabilize the monomeric form of the tau protein in order to maintain its function and shift the equilibrium from aggregates towards nontoxic monomers. We decided to use mirror-image phage display to generate all the D-enantiomeric peptides that are able to achieve the desired effect. Mirror image phage display allows the identification of all D-enantiomeric peptides as ligands for a given target, in this case the tau protein. In comparison to the normal, L-enantiomeric peptides, D-peptides are much more resistant to proteolytic degradation and are less immunogenic. These are important properties for potential therapeutics. It has been claimed that the aggregation of tau is dependent on two hexapeptides, namely ^306^VQIVYK^311^ (PHF6) and ^275^VQIINK^280^ (PHF6*) [12,13]. PHF6* is located on the second imperfect repeat and only present in tau isoforms that express the second imperfect repeat. The PHF6 region is located in the third imperfect repeat and is present in all tau isoforms. Both peptides are able to induce tau aggregation, but recent studies suggest that the PHF6* region has a stronger effect than the PHF6 region [14]. Cryo-electron microscopy of AD brains revealed that the core of PHFs contains the residues 306-378 of two identical protofilaments of tau [15]. Considering this, we decided to choose a 45-amino-acid-long fragment of tau as a target for mirror-image phage display. This target contained the amino acid residues of tau from 271 to 316. Thus, both the PHF6* and the PHF6 region were present in the fragment. We used the mirror image of tau(271-316) as a target for phage display selection and next-generation sequencing (NGS) for the analysis of the selection results. We successfully selected stable all D-enantiomeric peptides that formed large groups of homologous sequences enriched by the selection. Here, we show that the enriched peptides are able to stop tau aggregation in vitro and reduce the amount of tau fibril assembly in HEK293T cells.

## 2. Results

### 2.1. Peptide Selection Using Mirror-Image Phage Display and Next-Generation Sequencing

**Figure 1:**
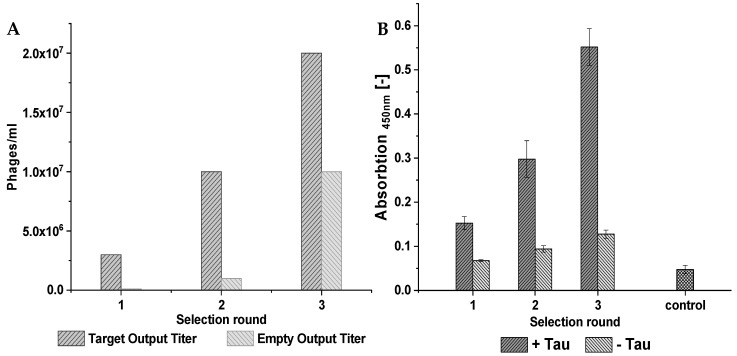
The selection progress was observed by following the output titer of target and empty selections as well as via enrichment ELISA. (**A**) For output titer determination, a phage dilution series was pipetted onto agar plates containing *E.coli K12*. The phage titer of the target and empty selections increased with each selection round. Target selection, depicted in dark grey, contained the fragment of tau while the empty selection, shown in light grey, did not contain any tau. The increase in total phage count after each round proves the enrichment of phages over the course of the selection. (**B**) The plate for the enrichment ELISA was coated with 20 pmol of D-tau(271-316), shown in dark grey, and was incubated with 5 × 10^10^ phages. Buffer was added instead of the D-tau(271-316) during coating to act as a negative control (light grey). Lastly, a separate control was performed to estimate the background noise level. This control did not contain any phages but was otherwise performed as the ELISA with D-tau(271-316). The signal intensity correlates with the amount of phages that are retained after several washing steps. We observed an increase in signal with higher selection rounds for wells that were coated with the D-tau(271-316), while the signal for the wells that were coated with buffer only showed a small increase. This indicates the enrichment of phages that are specific for the D-tau(271-316).

The peptide ligands that bind tau were obtained by mirror-image phage display selection against D-tau(271-316) to finally obtain all D-enantiomeric peptides that bind to and stabilize monomeric tau [16,17,18,19]. D-peptides are more stable and less immunogenic than their L-enantiomeric counterparts. Three rounds of selection were performed and the enrichment of the phages presenting peptides was estimated by observing the output titer of each round. Additionally, a second selection, the “empty selection”, was performed. This selection ran in parallel to the “target selection” and was later used as a negative control. The “empty selection” did not contain D-tau(271-316) on the surface of the selection. We observed an increase in the output titer by about 100% with each selection round for both selections (Figure 1A). The output titer of the target selection was always higher than the output titer of the empty selection, as was expected due to the enrichment of the phages that bind the D-tau(271-316). 

The average affinity of the phages from each selection round for the D-tau(271-316) was analyzed via enrichment ELISA. The results of the ELISA are displayed in Figure 1B. The ELISA started with an average initial signal of 0.15 for the wells containing D-tau(271-316) and 0.07 for the wells without D-tau(271-316). The signal for the wells containing the D-tau(271-316) doubled after the second selection round and again after the third and final selection round. The wells that were not coated with D-tau(271-316) showed only a small increase in signal intensity. The difference in signal intensity increased with each selection round. While the difference was around a factor of 2 in the first round, the difference increased to about 4.5 times in the third selection round. This experiment was also repeated with D-tau(271-316) concentrations of 60 pmol and 200 pmol coated on the plate used during the ELISA (Supplementary Figure S1). A highly similar increase in signal intensity, as described above, was observed. The measured signal intensity during the ELISA was proportional to the amount of bound phages that were retained in the wells after multiple washing steps. 

### 2.2. NGS Data Analysis Reveals Highly Conserved Peptide Clusters 

**Figure 2:**
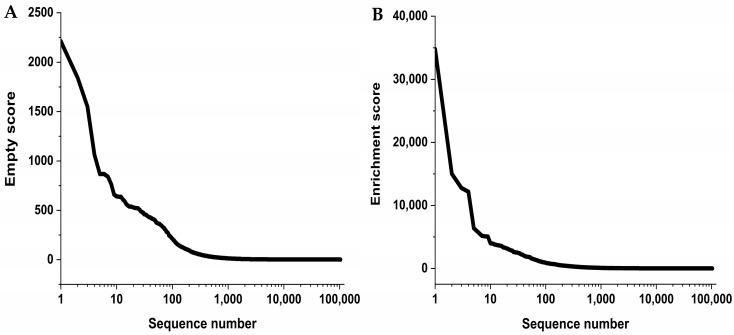
The selection yielded a small number of sequences with high empty or enrichment scores. The displayed distributions cover all sequences selected in a logarithmic scale on the x-axis. (**A**) After filtering with TSAT, every sequence was assigned an empty score. This score describes how much higher the frequency of a sequence of the last selection round is compared to its frequency within the control selection (empty selection). With the increasing empty score, the probability of a sequence to be target-specific rises as it is mainly enriched in the target selection. For this selection, we observed a minority of sequences having a very high empty score. Only sequences with an empty score of at least 250 were considered for further testing. (**B**) As with the empty score, an enrichment score was calculated and assigned to each sequence after filtering. This score describes the increase in frequency a sequence of the last target selection had compared to the library. The distribution of the enrichment score was very similar to the empty score, with a minority of sequences possessing a high score. Only sequences with an enrichment score of at least 250 were considered for further testing.

NGS features the ability to sequence millions of phage genomes coding for peptides presented on the phages. Every sequenced selection round generated around 1 million reads. One read consisted of approximately 200 nucleotides. Further processing of the generated NGS data was needed for analysis as only 48 out of the 200 nucleotides code the randomized peptide. The phage DNA sequence was processed with the tool TSAT [20]. The resulting amino acid sequences were filtered and subsequently clustered with the software Hammock [21]. The sequences were filtered according to their frequencies in the target selection compared to the empty selection and the direct control. Only the sequences that were enriched from one target selection round to the next one and that were also equal or less frequent in the empty selections and direct controls of the respective selection round passed the filter. Additionally, the empty and enrichment scores were calculated for the filtered sequences, and the peptides were valued according to their empty and their enrichment scores as well as the size of the generated clusters. After filtering, 100,898 unique sequences remained. These sequences were present in different abundances, with most of them appearing just once. When summed up, the total number of reads after filtering was 632,659. The parameters for Hammock were chosen to be standard parameters and resulted in the formation of highly conserved clusters that were used to generate position-specific scoring matrixes (PSSMs). A graphical illustration of the empty and the enrichment score distribution is shown in Figure 2. 

The highest enrichment score a sequence obtained was 34,807 (Figure 2B). This means that compared to the frequency in the library this sequence was 34,807 times more frequent in the last TS round. The empty score described in Figure 2A, on the other hand, is important in the differentiating of the sequences that are target-specific from the sequences that are not. It provides information on how much higher the frequency of a sequence was in the target selection compared to the empty selection. An ideal sequence should exhibit a high enrichment score and a high empty score and should form a large cluster of similar sequences after Hammock analysis. Only sequences with empty and enrichment scores of at least 250 were considered for further testing.

Due to their empty score, enrichment score, or the cluster size and quality they formed, ten all D-enantiomeric peptides (compounds) were chosen from the filtered sequences for further testing. Remarkably, in target selection round three, all of the highly enriched sequences were able to form their own clusters with highly conserved motifs. These ten compounds taken together equaled 54.25% of the total amount of reads. The compounds were named TF2D-1 to TF2D-10 (tau fragment 2 binding D-peptide 1 to 10). We concluded that these compounds had the highest chance to inhibit tau aggregation by stabilizing its monomeric conformation.

### 2.3. Screening via ThT Assay Revealed Two Compounds with Aggregation Influencing Properties

**Figure 3:**
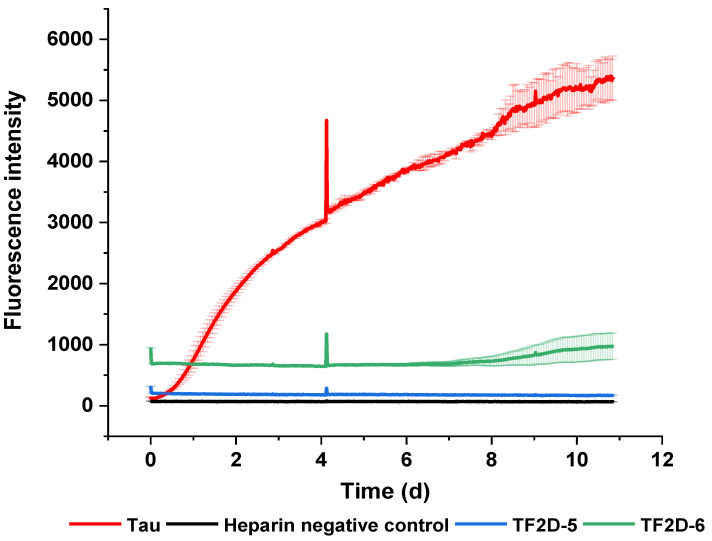
Screening for aggregation-inhibiting compounds via ThT assay. Each of the initial ten compounds was tested in a ThT screening to check if they had an aggregation-modifying effect on full-length tau 441. All compounds were mixed in a concentration of 50 µM with 50 µM recombinant full-length tau 441, 12.5 µM heparin, 500 µM DTT, 2% DMSO, and 20 mM ThT. Every well was adjusted to 100 µL with 1× PBS pH 7.4. Measurements were taken in intervals of 20 min. The plate was incubated at 37 °C and shaken for 15 s at 300 rpm before each measurement. The tau control did not contain any compounds, while the heparin negative control lacked both heparin and compounds. We chose 50 µM tau to allow de novo aggregation of tau to happen within days. We are fully aware that this is not a physiological concentration of tau. At about four days incubation time, measurement was prematurely terminated. Data recording was restarted without any time delay. The initial measurement points contained artefact signals that led to the spikes observed at about 4 days incubation.

Synthetically produced D-enantiomeric compounds were tested for their potential to stop the conversion of monomeric tau into fibrils. For this purpose, full-length tau 441 was used. The compounds were tested for a total duration of eleven days. In Figure 3, the two compounds with the highest shift in lag time are depicted.

As shown in Figure 3, the compounds TF2D-5 and TF2D-6 had a significant influence on the ThT-active aggregation of full-length tau 441 with heparin. Both compounds delayed the start of aggregation by at least seven days, while the addition of TF2D-5 completely inhibited ThT-active aggregation during the span of the experiment. From this, we concluded that both compounds are likely able to modify the aggregation pattern of full-length tau 441. If this effect was due to an interaction between tau and the compounds, which kept tau in its monomeric form, or if the interaction produced amorphous aggregates, it could not be recognized by ThT fluorescence and was addressed in further experiments. 

The initial ThT screening was further analyzed by the pooling of all the replicates of each sample, centrifuging them, and analyzing the resulting pellet and supernatant by SDS-gel electrophorese. The bigger aggregates would gather in the pellet after centrifugation, while the monomeric tau and the smaller aggregates would be retained in the supernatant. This allowed for a rough estimation of whether the compounds only affected the binding of ThT to the aggregates or whether the amount of bigger aggregates was reduced. It also served as an indicator for the formation of amorphous aggregates. For the samples containing the compounds TF2D-5 and TF2D-6, no visible pellet formed after centrifugation. Similarly, the amount of tau in the pellet fraction for the samples with these compounds was reduced compared to the samples without the compounds (see Supplementary Figure S2). Consequently, the amount of soluble tau contained in the supernatant fraction was slightly increased. From this, we concluded that the effectivity of both compounds in the suppression of ThT-active aggregation was not due to formation of ThT-negative amorphous aggregates. 

### 2.4. Lead Candidate TF2D-5 and Its Derivates

**Figure 4:**
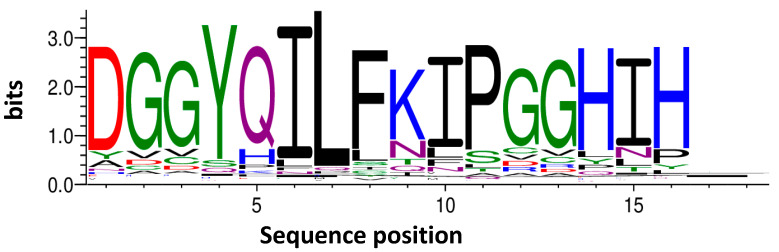
Position-specific scoring matrix of the TF2D-5 cluster. After analysis of the NGS data as well as the preliminary screening experiments, the D-peptide TF2D-5 and its derivate TF2D-5a were selected as lead candidates for the development of a therapeutic agent against tauopathies. Out of more than 600,000 sequences that were able to pass the filter, TF2D-5 amounted to about 3% of all sequences. The shown PSSM was generated with the Hammock program and was derived from 1970 different sequences that were obtained during selection. Letter size corresponds to the frequency with which an amino acid was present at the depicted position in the PSSM. These sequences were all enriched with different intensities, and the biggest letters for each position formed the exact sequence of TF2D-5 after clustering. This led to the decision to use the D-peptide with the sequence dGGyqilfkipGGhih for future experiments. TF2D-5a is TF2D-5 with five additional D-arginine residues at the C-term: dGGyqilfkipGGhihrrrrr.

Considering these findings and the higher lag time shift of TF2D-5 during the ThT experiments we decided to use TF2D-5 as a lead candidate. TF2D-5 was the fifth most abundant D-peptide sequence after TSAT filtering. It amounted to about 3% of all the sequences and had an empty score of 363.14 and an enrichment score of 257.79. It formed a cluster of 1970 unique, highly conserved sequences, as shown by the clustering with the Hammock software. The sequence of TF2D-5 is dGGyqilfkipGGhih. Figure 4 shows a position-specific scoring matrix (PSSM) of the TF2D-5 cluster. 

TF2D-5a is a derivate of TF2D-5. It features five additional arginine residues at the C-term (dGGyqilfkipGGhihrrrrr). The addition of arginine residues increases the solubility of TF2D-5a and has been shown to increase the membrane permeability of compounds [22]. Additionally a control D-peptide (CP1) was designed and ordered. This D-peptide features the same amino acid residue composition as TF2D-5 but has a randomized sequence (GiiGpqykdhGhfliG). It was used in further experiments as a control to exclude the effects due to a certain amino acid composition rather than the specific sequence. 

### 2.5. TF2D-5 and TF2D-5a Inhibit Tau Aggregation during ThT Assay

**Figure 5:**
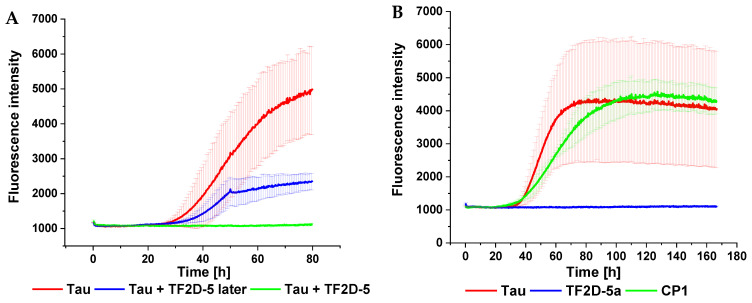
Tau aggregation-influencing effect of TF2D-5 and TF2D-5a. The ThT assay was performed in a FLUOstar plate reader from BMG Labtech. Twenty-five micrometers recombinant full-length tau 441 was mixed with 12.5 µM heparin, 200 µM DTT, 0.05% NaN_3_, and 20 mM ThT. Compounds were added with a concentration of 25 µM. The total volume was adjusted to 100 µL with PBS pH 7.4, and the plate was measured with bottom optics. The experiment ran at 37 °C with measurement intervals of 20 min. Before each measurement, the plate was shaken for 15 s at 300 rpm. A 3 mm borosilicate bead was added to each well. (**A**) The effect of TF2D-5 when added at different time points during the ThT assay. When added at the start of the experiment no aggregation can be observed for the duration of the experiment (green). When added during the exponential phase at time point 48 h, the aggregation slows immediately and then stops (blue). (**B**) The addition of TF2D-5a to the sample inhibits tau aggregation during the complete experiment which equals a lag time increase of at least 400% (blue). The addition of control D-peptide 1 (CP1) did not exhibit the same effect as the addition of the other compounds (green). CP1 was obtained by randomizing the amino acid residue composition of TF2D-5.

Both compounds were repeatedly tested for their ability to modify the ThT-active aggregation of full-length tau 441 during the ThT assays. In addition to the effect of TF2D-5a on ThT-active tau aggregation, we also tested the effect of TF2D-5 addition to an ongoing aggregation as well as the effect of the control D-peptide CP1. The results of these assays were consistent and are presented in Figure 5. 

The data illustrate that the presence of TF2D-5 or TF2D-5a in a 1:1 molar ratio at the start of the experiment had a tremendous effect on the lag time of the tau aggregation. In the case of TF2D-5, no aggregation was observed for the duration of the experiment. Even a delayed addition of TF2D-5 during the exponential aggregation phase of tau impeded further aggregation (Figure 5A). Although the blue and red curves in Figure 5A are not identical up to the time point of the peptide addition, they appear within each other’s margins of error and are thus not significantly different. The curve progression upon peptide addition, however, compared to the experiment without peptide, is significantly reduced, which indicates decelerated fibrillation upon peptide addition. The addition of TF2D-5a resulted in the complete inhibition of aggregation during the time of the experiment. The addition of control D-peptide one (CP1) had no visible effect on the lag time of the full-length tau 441 aggregation (Figure 5B). From this, we concluded that both TF2D-5 and TF2D-5a were able to significantly increase the lag time of the full-length tau 441 aggregation. We also concluded that the effectivity of TF2D-5 and TF2D-5a was sequence-specific as the control D-peptide CP1 was not able to produce similar results, despite having the same amino acid residue composition as TF2D-5. Finally, we could show that it was possible to achieve an aggregation-inhibiting effect even during ongoing aggregation by providing TF2D-5.

### 2.6. TF2D-5 and TF2D-5a Inhibit Full-Length Tau Aggregation Substoichiometrically

**Figure 6:**
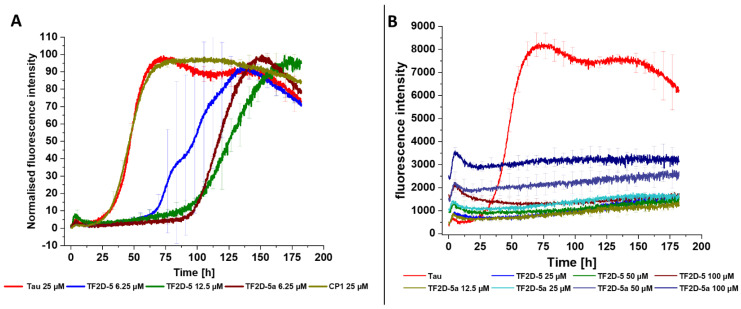
ThT experiment with sub-stoichiometric D-peptide concentrations. (**A**) Normalized summary of all peptide concentrations that did not completely inhibit tau aggregation during the experiment. Concentration of full-length tau was 25 µM. Addition of 6.25 µM of TF2D-5 resulted in an average lag time increase of about 30 h or 100%, while 12.5 µM shifted the average lag time by about 60 h or 200%. Addition of 6.25 µM of TF2D-5a increased the average lag time by about 60 h or 200%. CP1 in a concentration of 25 µM had no effect on the lag time. (**B**) Non-normalized summary of all peptide concentrations that fully inhibited tau aggregation for the duration of the experiment.

To investigate whether TF2D-5 and TF2D-5a induce an extension of the lag time of tau aggregation, we performed a ThT experiment with sub-stoichiometric D-peptide concentrations. For this, we used tau that was produced according to the protocol in the Section 4. Twenty-five micrometers of full-length tau was incubated with 6.25 µM, 12.5 µM 25 µM, 50 µM, and 100 µM of D-peptides. As expected, we observed an increase in the lag time of the full-length tau aggregation with the increasing D-peptide concentrations. The addition of 6.25 µM of TF2D-5 resulted in an average lag time shift of about 30 h, which is a doubling of the lag time (100% increase). The TF2D-5 at 12.5 µM shifted the lag time by about 60 hours (a 200% increase). The addition of 6.25 µM of TF2D-5a shifted the lag time by about 60 h (a 200% increase). In contrast, 25 µM of the control peptide CP1 did not have any effect on the lag time (Figure 6). Every concentration of TF2D-5 and TF2D-5a that was higher than 12.5 µM inhibited tau aggregation until the end of the experiment, suggesting that both compounds act sub-stoichiometrically.

### 2.7. TF2D-5 Modified Fibril Growth/Atomic Force Microscopy Measurements

**Figure 7:**
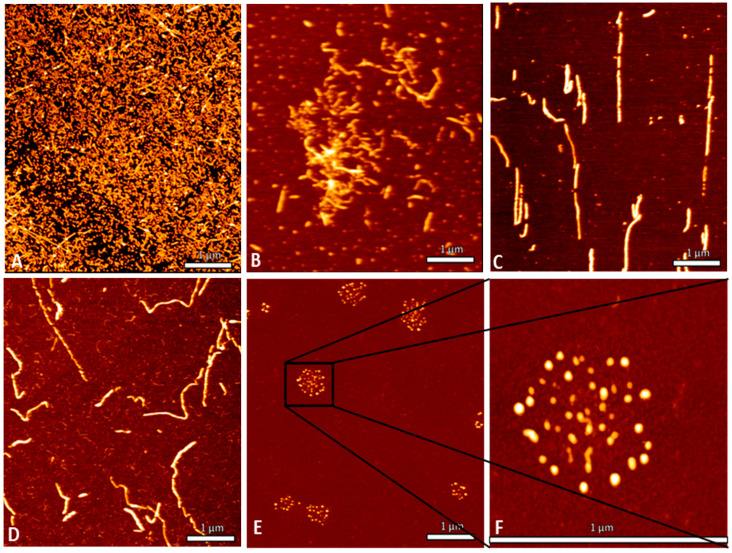
Atomic force microscopy (AFM) measurement using samples obtained after ThT assay with full-length tau 441 aggregation. All AFM measurements were performed by drying 1 µL on mica, followed by two washing steps with 100 µL water and measuring on JPK Nanowizard3 with intermittent contact mode on air and an OMCL AC160TS as a cantilever. The height scale was 0 to 10 nm for (**A**,**B**), 0 to 5 nm for (**C**,**D**), and 0 to 20 nm for (**E**,**F**). (**A**) Control aggregation of full-length tau 441 without any compounds. The whole mica was full of fibrils, and single fibrils were hard to differentiate. (**B**) One to ten dilution of 7A. After dilution, single fibrils became visible, and it was again confirmed that fibrils were present. (**C**) Addition of CP1 did not fully inhibit fibril formation. (**D**) TF2D-5 was added during the exponential phase of ThT-active tau aggregation. Here, the amount of tau fibrils that were found was less when compared to the control. (**E**) Addition of TF2D-5 at the start of the experiment. No fibrils were observed in these samples. Instead, clusters of spots were found throughout the mica, most probably resembling drying artifacts. (**F**) Enlarged representation of (**E**).

We used atomic force microscopy (AFM) to visualize the effect of TF2D-5 addition to the aggregation of full-length tau 441 in a ThT assay. For this purpose, we harvested the ThT samples (Figure 5) after the experiment and analyzed them. The initial concentration of full-length tau 441 without the addition of the compounds was too high to identify individual fibrils (Figure 7A). It was thus diluted in a 1:10 ratio. The corresponding AFM picture is depicted in Figure 7B and shows the fibrils which were mostly intact. Figure 7C represents the undiluted samples that were incubated with the control D-peptide CP1 during the ThT assay. The addition of TF2D-5 during the exponential phase of the aggregation yielded fewer total fibrils compared to the samples without TF2D-5. Figure 7E,F present the effect that was achieved by the TF2D-5 that had already been added at the start of tau ThT aggregation. Here, no fibrils were observed. The clusters of small spots observed here are most probably drying artifacts. Considering these results and comparing them with the results from the ThT assay, we conclude that the addition of TF2D-5 results in a significant deceleration in in vitro tau fibril formation.

### 2.8. TF2D-5 and TF2D-5a Exhibit Nanomolar Affinity for Tau

**Figure 8:**
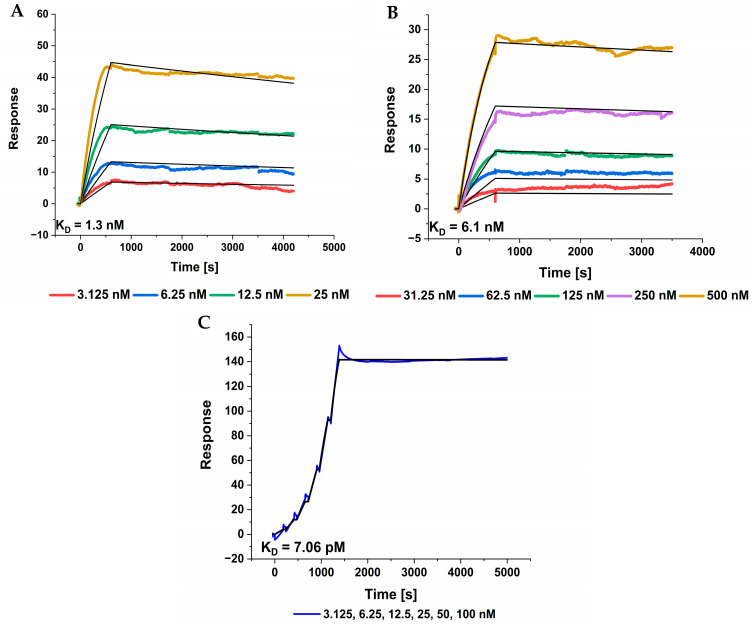
SPR measurements of TF2D-5 and TF2D-5a with monomeric full-length tau 441 and tau repeat region 3. (**A**) Binding curves and calculated fit for the affinity of TF2D-5 for monomeric full-length tau 441 resulted in a K_D_ of 1.3 nM. The concentration of tau during the measurement ranged from 3.125 nM to 25 nM. (**B**) Binding affinity of TF2D-5a for monomeric full-length tau 441 was determined to be 6.1 nM. Tau was used in concentrations ranging from 31.25 nM to 500 nM. SPR measurements of A and B were recorded on a T200 Biacore instrument, the corresponding compound was immobilized on a CM5 chip from Cytiva while monomeric tau served as analyte. Tests were performed with TBS pH 7.4, 0.05% Tween20 and 10 µM DTT as running buffer. Fit calculation was completed with the internal evaluation software of the T200 Biacore. A 1:1 model was used for both fits. (**C**) Single cycle kinetic measurement of TF2D-5a to the R3 domain of tau resulted in a K_D_ of 7 pM. The concentrations for the single cycle measurement ranged from 3.125 nM to 100 nM. Measurements of C were performed on a Biacore 8K using a CM5 chip from Cytiva. TF2D-5a was immobilized on the chip while the R3 region of tau was used as analyte. PBS pH 7.4 with 0.05% Tween20 as used as running buffer. Fit calculation was performed using the Biacore insight evaluation software and a 1:1 model. Applied tau concentrations for TF2D-5 and TF2D-5a have been finally chosen based on preliminary experiments and the estimated K_D_ values obtained thereof. We opted to measure in multicycle measurements, where possible (**A**,**B**). For (**C**), this was not possible due to the extremely low off rate.

The binding constants of TF2D-5 and TF2D-5a to full-length tau 441 were determined with SPR spectroscopy. For this purpose, both compounds were immobilized on the surface of different SPR chips, while full-length tau 441 was injected as an analyte. The calculated K_D_ values for both compounds were determined by a 1:1 fit model and resulted in 1.3 nM (Figure 8A) and 6.1 nM (Figure 8B) for TF2D-5 and TF2D-5a, respectively. Especially noteworthy in this case was the slow dissociation that was observed during the measurement. The calculated dissociation rate (kd) for both compounds was 1.9 × 10^−5^ s^−1^ (Figure 8A) to 4.3 × 10^−5^ s^−1^ (Figure 8B). In another experiment, we measured the affinity of TF2D-5a towards the different repeat domains of tau (Figure 8C). We observed the strong binding of TF2D-5 to the R3 domain but not to the other repeat domains. The measured curves and the calculated fit are shown in Figure 8. TF2D-5a was selected for binding to tau(271-316). The strongest binding, however, was observed for tau(304-336). Interestingly, the apparently non-overlapping region of tau(271-303) has a very high sequence identity with tau(317-336). This result points to two potential conclusions. First, the sequence specificity of TF2D-5a is surprisingly high, given that the other repeat regions also have substantial sequence similarity. Second, the affinity of TF2D-5a for repeat 3 is about 1000 times higher than for full-length tau. This can only be explained if the other sequence regions of tau or a fraction of them somehow “protect” repeat 3 from intermolecular interactions. Indeed, recent work describes intermolecular interactions within tau. Some of them are reduced in liquid–liquid phase separation, promoting tau aggregation [23]. Such intermolecular interactions do not need to be of high affinity in order to affect the differential binding of TF2D-5a to repeat 3 vs. full-length tau because of the high local concentrations of intramolecular tau regions. This is of course just a speculation that would explain the observed pM affinity of TF2D-5a for tau repeat 3 and the nM affinity for full-length tau. The observation certainly deserves more investigation in the future.

### 2.9. MST Measurements Exhibit High Affinity of TF2D-5 and TF2D-5a towards Tau

**Figure 9:**
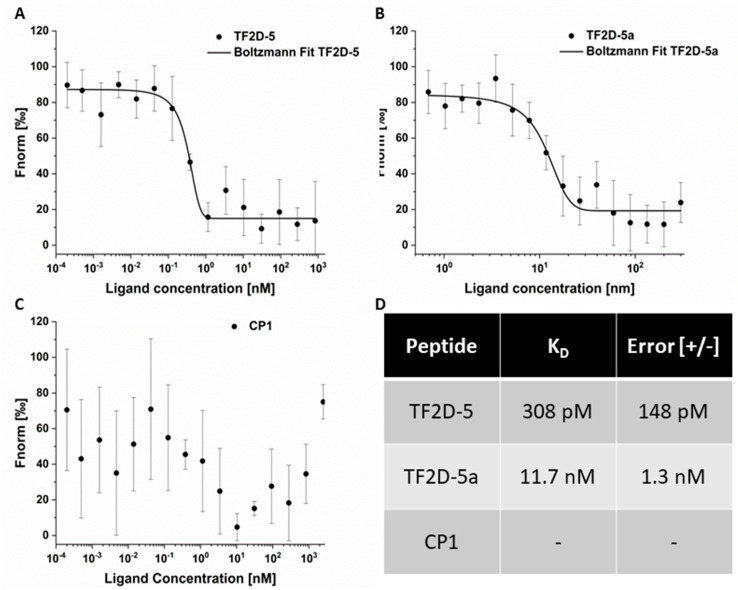
MST measurements of TF2D-5, TF2D-5a, and CP1 with full-length tau. (**A**) Boltzmann fit of TF2D-5 MST measurements with full-length tau. TF2D-5 was labeled with CF633. The concentration of TF2D-5 was 500 nM, while the concentration of full-length tau varied between 2.5 µM and 0.2 pM. The measurement was performed in 1× PBS pH 7.4 containing 150 mM NaCl. (**B**) Boltzmann fit of TF2D-5a MST measurements with full-length tau. TF2D-5a was labeled with CF633. The concentration of TF2D-5a was 62.5 nM, while the concentration of full-length tau varied between 300 nM and 0.5 nM. The measurement was performed in 0.1 M sodium phosphate buffer pH 7.4 containing 75 mM sodium sulfate and 0.05% Tween20. (**C**) Average of CP1 MST measurements with full-length tau. CP1 was labeled with CF633. The concentration of CP1 was 62.5 nM, while the concentration of full-length tau varied between 2.5 µM and 0.2 pM. The measurement was performed in 0.1 M sodium phosphate buffer pH 7.4 containing 75 mM sodium sulfate and 0.05% Tween 20. (**D**) Calculated K_D_s of each peptide and full-length tau. TF2D-5 has a K_D_ of 308 pM with an error of 148 pM to full-length tau, which is tenfold lower that the K_D_ determined by SPR measurements. TF2D-5a has a K_D_ of 11.7 nM with an error of 1.3 nM. This is close to the K_D_ determined by SPR measurements (6.1 nM). For CP1, no fit could be performed, and all measurements indicated no binding in the tested concentration range. All experiments were repeated four times and the average of all measurements was calculated, which in turn was used to calculate the fit (Boltzmann).

To verify the high affinity and to rule out potential effects of the immobilization on the binding affinity, we carried out MST measurements of the fluorescent labelled peptides and full-length tau (Figure 9). Here, we were able to observe the interaction without immobilization on a surface. Our measurements yielded a K_D_ of 11.7 nM for TF2D-5a, which is only slightly different from the SPR-obtained K_D_ of 6.1 nM. For TF2D-5, we obtained a K_D_ of 308 pM, which was tenfold lower than our previous result (1.3 nM). We also measured the affinity of our control peptide CP1 towards full-length tau and demonstrated that there is no affinity in the same concentration range as TF2D-5. We therefore concluded that the observed effects were sequence-specific.

### 2.10. TF2D-5 and TF2D-5a Preserve Monomeric Tau in Sub-Stoichiometric Concentrations during Seeded Aggregation

**Figure 10:**
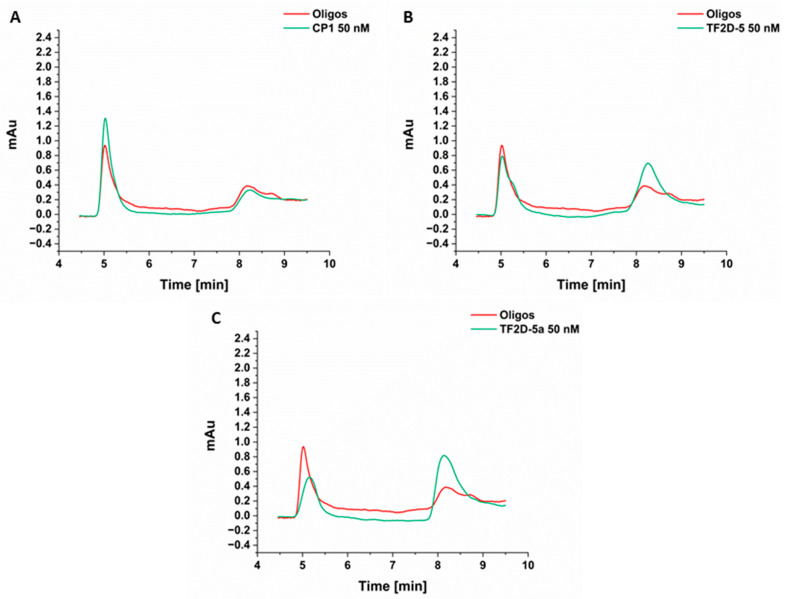
HPLC measurements after seeded aggregation of tau with soluble tau oligomers. Tau monomer with a concentration of 100 nM was incubated with 100 nM soluble tau oligomers and variable sub-stoichiometric concentrations of TF2D-5, TF2D-5a, and CP1. (**A**) CP1 was added at a concentration of 50 nM to the sample. No deviation from the reference sample was observed. (**B**) TF2D-5 was added at a concentration of 50 nM to the sample. We observed an increase in the retained tau monomer peak (8–8.5 min) with increasing concentrations of TF2D-5. (**C**) TF2D-5a was added at a concentration of 50 nM. We observed a stronger increase in the retained monomer peak than with TF2D-5. Additionally, we observed that the oligomer peak decreased in height.

The most likely result of TF2D-5 and TF2D-5a binding towards monomeric tau that can be expected is in an equilibrium shift from aggregated tau towards monomeric tau. To investigate this, we performed a seeded aggregation assay with 100 nM of monomeric tau and 100 nM of soluble tau aggregates as seeds, in the presence and absence of TF2D 5, TF2D-5a, and the control peptide CP1 (Figure 10A–C). The samples were incubated for 96 h to ensure the occurrence of sufficient aggregation, and the samples were subsequentially analyzed via size exclusion chromatography. Figure 10 shows the respective chromatograms obtained without any D-peptide (red) and those obtained in the presence of 50 nM of TF2D-5 (Figure 10A), TF2D-5a (Figure 10B), and CP1 (Figure 10C). The monomeric tau elutes started at 8 min and the soluble oligomer elutes after around 5 min. While the addition of CP1 during seeded aggregation had no effect, the presence of TF2D-5 and TF2D-5a increased the amount of retained monomeric tau.

### 2.11. TF2D-5 and TF2D-5a Were Not Toxic to SHSY5Y and PC12 Cells

A very important property of a therapeutic agent is its tolerability for cells. As a very rough estimation, we measured the effect of different TF2D-5 and TF2D-5a concentrations on SHSY5Y and PC12 cells. From the data, we can conclude that both TF2D-5 and TF2D-5a are not toxic to the used cell lines. This was true for all the tested concentrations (Supplementary Figure S3).

### 2.12. Blood–Brain Barrier Permeability

**Figure 11:**
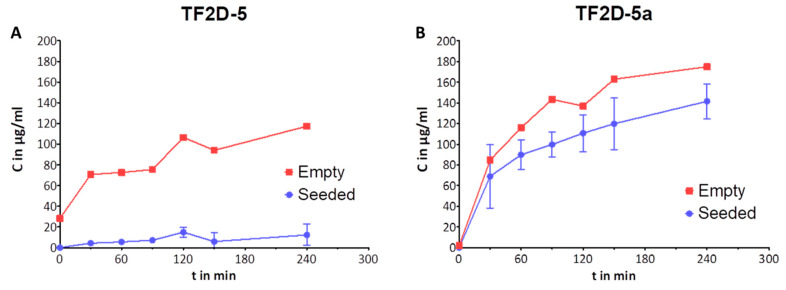
In vitro blood–brain barrier assay for TF2D-5 and TF2D-5a. An amount equaling 1 mg/mL TF2D-5 (**A**) or TF2D-5a (**B**) was added to an insert that was seeded and covered with endothelial and astroglial cells, representing the in vitro blood–brain barrier, or an empty insert serving as a control. After different time points, samples were taken and analyzed by quantitative HPLC analysis. Data given as mean ± SD out of three independent replicates.

The ability to cross the blood–brain barrier is one of the most important skills that a potential therapeutic agent for neurodegenerative diseases may possess. To evaluate whether the compounds TF2D-5 and TF2D-5a exhibit this ability, we performed an experiment with an in vitro blood–brain barrier model. Each peptide was tested at different time points in triplicate next to an unseeded negative control (“Empty”) and quantitatively analyzed via HPLC using a calibration curve to determine the concentration of the peptide in the receiving chamber at each point in time (Figure 11). From these values, the volume of each chamber, and the respective time, a P_app_ permeability coefficient in cm²/s was calculated for each substance to represent its ability to cross the in vitro blood–brain barrier model. A P_app_ value greater than 1 × 10^−5^ would indicate an especially good permeability, while substances that do not cross the barrier well reach values of as low as 1 × 10^−7^. Caffeine, a substance known to easily pass the blood–brain barrier, reaches a P_app_ value of 3.18 × 10^−5^ in our model, while Fluorescein, an established paracellular marker, has a P_app_ value of 8.86 × 10^−7^. We obtained P_app_ scores of 3.6 × 10^−6^ for TF2D-5 and 4.8 × 10^−5^ for TF2D-5a. The results show that both compounds were able to cross the model blood–brain barrier.

### 2.13. TF2D-5 and TF2D-5a Are Very Stable in Different Body Fluids

**Figure 12:**
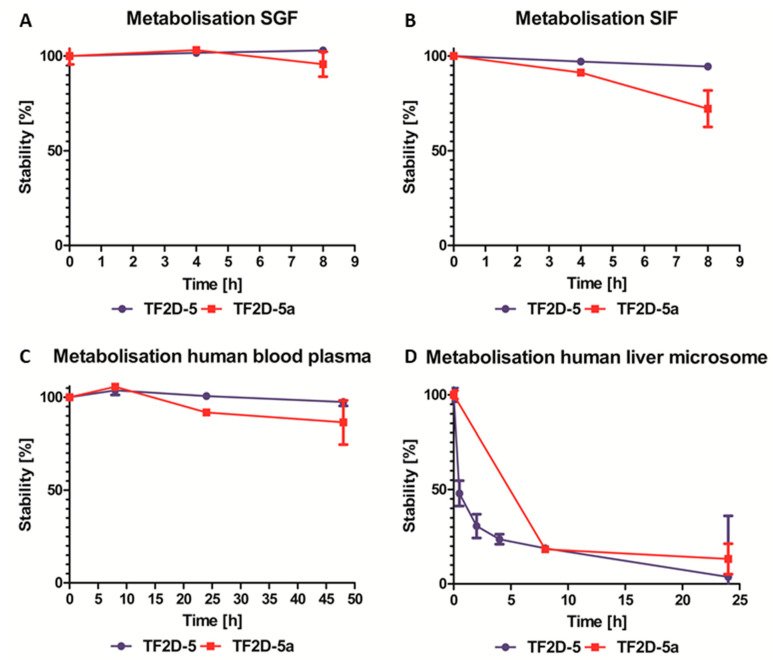
Stability assay of TF2D-5 and TF2D-5a using different simulated human body fluids. (**A**) Stability of compounds in simulated gastric fluid (SGF). The simulated gastric fluid consisted of 2 mg/mL sodium chloride, 3.2 mg/mL pepsin, and 80 mM HCl; pH 1. (**B**) Stability of TF2D-5 and TF2D-5a in simulated intestinal fluid (SIF). Simulated intestinal fluid consisted of 6.8 mg/mL potassium dihydrogen phosphate, 10 mg/mL pancreas powder, and 15.4 mM NaOH; pH 6.8. (**C**) Stability of TF2D-5 and TF2D-5a in human blood plasma. K3EDTA human blood plasma was purchased from BioTrend. (**D**) Stability of TF2D-5 and TF2D-5a in human liver microsomes. Human liver microsomes consisted of 6 mg/mL protein (containing Cytochrome P450 (CYP) enzymes), 75 mM sucrose, 1.3 mM NADPH, 3.3 mM D-glucose-6-phosphat, 0.2 u/mL D-glucose-6-phosphat dehydrogenase, 3.3 mM magnesium chloride, and 100 mM potassium phosphate; pH 7.4.

Another very important quality of therapeutic compounds is their stability once they are introduced into the human body. As mentioned above, the stability of all D-enantiomeric compounds is increased in the human body compared to all L-enantiomeric compounds. To assess how stable TF2D-5 and TF2D-5a are, if taken orally, we performed stability assays. Both compounds were tested using simulated gastric fluid (SGF, Figure 12A), simulated intestinal fluid (SIF, Figure 12B), human blood plasma (Figure 12C), and human liver microsomes (Figure 12D). We observed that TF2D-5 and TF2D-5a were very stable in simulated gastric fluid. After 8 h, most of the initial amount was not remarkably decreased. While TF2D-5 was stable in simulated intestinal fluid, we observed a decrease in the TF2D-5a level after about 8 h, where the TF2D-5a levels decreased by 27.8%. When incubated in human blood plasma, both TF2D-5 and TF2D-5a were stable. After 48 h, 97.4% of TF2D-5 and 86.5% of TF2D-5a were still detectable. The compounds were metabolized fast in liver microsomes. Here, only 3.7% of TF2D-5 and 13.2% of TF2D-5a were still unmetabolized after 24 h. The possible metabolizations include degradation of the compounds, redox reactions, and other reactions supported by the CYP450 enzymes [24].

### 2.14. TF2D-5a Inhibits Tau Aggregation in HEK293T Cells

**Figure 13:**
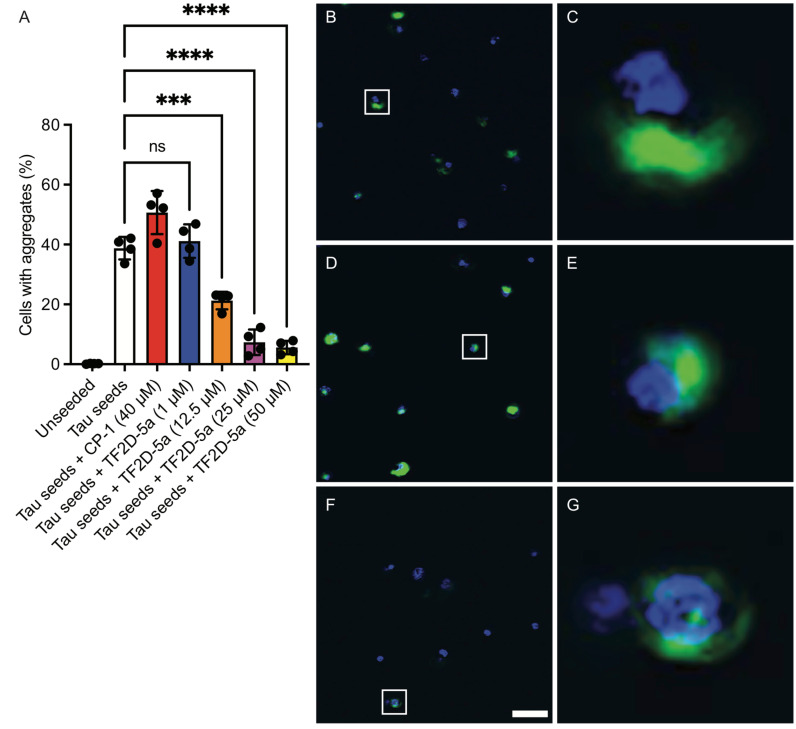
TF2D-5a inhibits tau aggregation in cells. In order to compare the abiliy of TF2D-5a and CP-1 to inhibit tau aggregation without interference from potentially different cell permeabilities, we used lipofectamine for cell transfection, as described in the Section 4. (**A**) TF2D-5a and the negative-control D-peptide CP-1 were tested for their ability to inhibit tau aggregation in tauK18(LM)-YFP cells. “ns” abbreviates the expression “non significant”. (**B**) In contrast to unseeded cells, which did not harbor any tau aggregates at day three after plating, seeding with sonicated tau fibrils induced aggregation in approximately 40% of cells after three days of incubation. (**D**) A three-day treatment with the negative-control D-peptide CP-1 did not inhibit tau aggregation. (**F**) In contrast, treatment with increasing concentrations of TF2D-5a led to a concentration-dependent reduction in the number of cells with tau aggregates, which was significant at 12.5 µM and further reduced tau aggregation at 25 µM and 50 µM TF2D-5a. Panels (**C**,**E**,**G**) represent magnifications of cells indicated by white rectangles in panels (**B**,**D**,**F**), respectively. The scale bar represents 50 µm for panels (**B**,**D**,**F**), and 5 µm for panels (**C**,**E**,**G**). Significance was calculated using one-way ANOVA followed by Dunnett’s multiple comparisons test. Three asterisks denote a *p* value less than 0.001 and four asterisks a *p* value less than 0.0001.

To further verify whether the inhibitory effect of TF2D-5a on tau aggregation is limited to cell-free systems or is also valid in a cellular environment, TF2D-5a was transfected together with tau seeds in tauK18(LM)–YFP-expressing HEK293T cells. These cells stably express the four repeat domains of human tau with the familial P301L and V337M mutations fused to YFP, which yields a weak diffuse green fluorescence in the cytosol. Tau aggregation can be induced in these cells after seeding with sonicated tau fibrils through transfection and can be detected as green foci with high fluorescence intensities by automated live-cell imaging. Similar cell assays for seeded tau aggregation have been described by other groups and demonstrate that synthetic and patient-derived tau seeds induce protein aggregation in cells after 3–12 d of incubation [25,26,27]. We imaged the cells after incubating them for 3 d either without or with sonicated tau fibrils as seeds and in the presence or absence of D-compounds (Figure 13). While the unseeded cells did not accumulate any tau aggregates, approximately 40% of the seeded cells accumulated tau aggregates (Figure 13B). An ineffective control D-peptide (CP-1), with the same amino acid composition, except for the five additional arginine residues, but a different distribution of the amino acids to that of TF2D-5a and no affinity for monomeric tau, was unable to inhibit tau aggregation after seeding (Figure 13C). However, TF2D-5a showed a concentration-dependent reduction in aggregate-positive cells when used at a concentration of 12.5 µM and above (Figure 13D). These results show that the previously observed inhibitory effect of TF2D-5a on the capacity of tau seeds to induce aggregation is also valid in a cellular environment under strong seeding conditions.

## 3. Discussion

Most of the currently developed therapeutic agents for Alzheimer’s disease and other tauopathies focus on either the elimination of toxic oligomers of aggregated tau or its expression downregulation. Up to now, there is no approved therapeutic agent that effectively targets tau pathology. As recent studies show that the onset of symptoms during Alzheimer’s disease correlates better with tau pathology rather than Aβ, it may be more efficient to target the tau protein [10]. Therefore, the approach of stabilizing and protecting the monomeric form of tau, while shifting the aggregation equilibrium towards its monomeric conformation and destabilizing aggregates, may prove to be a successful strategy. The use of mirror-image phage display to generate all D-enantiomeric compounds has already been shown to yield all D-peptides which are able to bind tau aggregates [28,29,30]. An all D-enantiomeric peptide that is able to bind and stabilize monomeric tau does not yet exist.

In this study, we demonstrated the successful selection of the all D-enantiomeric compound TF2D-5 and its derivative TF2D-5a, which are able to bind monomeric tau with a nanomolar K_D_ and to inhibit the formation of tau aggregation in vitro and in cells. We were able to identify these compounds by using mirror-image phage display combined with NGS, which allowed for the in-depth analysis of the selections.

A crucial property of selected compounds as potential therapeutic agents is the inhibition of tau aggregation. The ability of TF2D-5 and TF2D-5a to inhibit tau aggregation in vitro was studied with different methods. ThT assays were used to observe changes in the lag time and the fluorescent levels, while gel electrophoresis and AFM measurements were used to visualize the effects of both compounds and to ensure that no amorphous aggregates were formed. All of the obtained results indicate that both compounds have a stabilizing effect on monomeric tau. The ThT assays with the addition of either TF2D-5 or TF2D-5a revealed an increased lag time of tau aggregation, while the addition of TF2D-5 to an ongoing tau aggregation was able to stop its aggregation from the point of introduction. These observations were also consistent with the gel electrophoresis results. The presence of TF2D-5 lowered the amount of tau that sedimented by centrifugation compared to the samples that did not contain TF2D-5. The visualization of the tau aggregation inhibiting effectivity of the investigated compounds by AFM measurements revealed that the addition of TF2D-5 during the lag phase of aggregation led to no observable fibrils on the micas. Similarly, the addition of TF2D-5 during the exponential phase led to a reduction in overall fibril quantity. This might be due to the mode of fibril growth during tau aggregation. Recent studies showed that tau fibril size distribution is dominated by the breaking and linkage of tau fibrils where longer fibrils fracture and act as multiple seeds which elongate by monomer addition and form longer fibrils [31]. This elongation is disrupted by the TF2D-5 addition as it stabilizes the monomeric tau and prevents its recruitment towards smaller fibrils, resulting in broken fibrils. The sequence specificity of TF2D-5 was verified by the use of a control D-peptide, which features the same amino acid composition as TF2D-5 but in a randomized sequence. This randomized control D-peptide was ineffective for tau aggregation inhibition during the ThT assays.

High affinity towards the target is a key property. We used SPR measurements to determine the K_D_ of TF2D-5 and TF2D-5a. Both compounds possess high affinities towards the target, resulting in low K_D_s of 1.3 nM and 6.1 nM, respectively. In further experiments, we validated these K_D_s and found that the actual K_D_ of TF2D-5 towards monomeric tau is even lower, with 308 pM. Recent papers reported on the cryo EM structures of tau fibrils isolated from the PHFs of Alzheimer patients. They revealed that the R3 domain is part of the fibril core [15], thus binding within this region might destabilize the fibril structure of tau or lead to steric hindrance during the incorporation of monomeric tau with bound TF2D-5 and TF2D-5a. Although TF2D-5 has the higher affinity towards full-length tau (308 pM versus 11.7 nM), we observed that the aggregation inhibition efficiency of TF2D-5a is superior. We assume that the increased inhibition properties of TF2D-5a are due to the proposed binding site of TF2D-5. In Figure 8C, we show the high affinity of TF2D-5 towards the imperfect repeat R3 of tau. This imperfect repeat was shown to be essential for the aggregation of tau as it is incorporated into the core of the fibril during aggregation [15]. The binding of TF2D-5 towards this site might lead to steric hindrances during aggregation. The addition of five arginine residues to the peptide results in an elongated peptide that may reach further into the potential fibril core, resulting in a stronger aggregation destabilization. At the same time, this addition is not optimized for tau binding and results in a lower affinity towards the binding site. The expansion of the peptide with five arginine residues was supposed to increase its solubility, and it is suspected that this increases the peptide’s ability to cross the blood–brain barrier [22]. We believe that the affinity for tau could be increased by replacing the arginine residues with other amino acids.

Considering the data obtained in the monomer quantification, we were able to show that both peptides increase the amount of monomer that is retained during a seeded aggregation. This effect was shown to be present at sub-stoichiometric concentrations, and we hypothesize that this effect is even stronger in a de novo aggregation or with higher concentrations of peptide.

We have shown that the effect of TF2D-5 and TF2D-5a is sub-stoichiometric. This again raises the question of the mechanism by which the aggregation of tau is inhibited. We currently hypothesize that this behavior is related to the binding of monomeric tau by TF2D-5 and TF2D-5a. Considering the SPR results, we assume that the peptides bind in repeat region three of tau. This region was found to be incorporated into the fibril core of aggregated tau [15]. Binding in this region could destabilize the fibril structure or lead to a change in the energetically most favorable conformation, effectively protecting monomeric tau from conversion into fibrils. The ability of TF2D-5 and TF2D-5a to inhibit tau aggregation at sub-stoichiometric concentrations could come from the amount of “unprotected” tau that is available. A decrease in the amount of tau that can form fibrils may lead to a longer lag phase until this chance-based cascade is triggered. This would fit with the shown data where the addition of very low amounts of TF2D-5 and TF2D-5a increases lag time but does not inhibit fibril formation completely. Another explanation may be found in the structural changes that occur during oligomerization. It was shown that the microtubule binding regions of tau adapt a paperclip-like formation with the N-term and C-term in the monomeric form. This paperclip formation collapses during aggregation, exposing the potential binding site for TF2D-5 and TF2D-5a [23]. As shown in the SPR experiments, the affinity of the peptides towards this region is in the low picomolar range. TF2D-5 and TF2D-5a could bind to the exposed binding site and stop an ongoing conversion into oligomers due to the steric hindrances introduced due to the binding.

Moreover, as expected for all D-peptides, the metabolic stability of TF2D-5 and TF2D-5a in different simulated environments connected to the uptake and digestion of therapeutic agents was quite favorable. From our experiments, we conclude that both compounds might be stable in SGF, SIF, and human blood plasma. TF2D-5 showed a particularly high stability in SGF, SIF, and human blood plasma. Both compounds are more stable in human blood plasma than RD2, an all D-enantiomeric peptide selected against Aβ that recently successfully finished clinical phase I trials [32] and was also investigated for its metabolic stability [33]. TF2D-5 was not very stable in liver microsomes. After 24 h, only 3.7% of TF2D-5 and 13.2% of TF2D-5a were unmetabolized. Compared to other all D-enantiomeric compounds, this metabolization speed is fast [33]. Moreover, TF2D-5 and TF2D-5a may well cross the blood–brain barrier.

Lastly, TF2D-5a was able to reduce the amount of cells with tau aggregates by about 80% compared to tau aggregation in the absence of TF2D-5a or the presence of a control D-peptide without any affinity for tau.

## 4. Material and Methods

### 4.1. Mirror-Image Phage Display

The mirror-image phage display describes a technique to select D-peptide binders of L-target proteins from a library. This is conducted by using a D-target and selecting with the L-peptides presented on the phages. The phages that bind to the D-target are analyzed, and the chosen peptides are synthetically produced as D-peptides. These peptides are then analyzed for their ability to bind the target and are, in the case of successful binding, further developed.

### 4.2. Target

The target of the phage display selection was a custom designed all D-enantiomeric fragment of tau consisting of the amino acids 271-316 out of the full-length tau 441(D-tau271-316). It encompasses the imperfect repeat 2 and part of the imperfect repeat 3 of the full-length 2N4R tau protein. The hexapeptides PHF6 and PHF6*, which are important for tau aggregation, were present in the imperfect repeat 2 (PHF6*) and 3 (PHF6). Both regions were included in the target design as binding near these regions might stabilize monomeric tau. A biotin tag was added to the N-terminus. The C-terminus was amidated.

### 4.3. Mass Spectroscopy

Mass spectroscopy was performed at the Central Institute for Engineering, Electronics and Analytics (ZEA-3) within the Forschungszentrum Jülich to verify the identity of the mirror-image D-tau(271-316).

### 4.4. Selection

#### 4.4.1. Strategy

**Figure 14:**
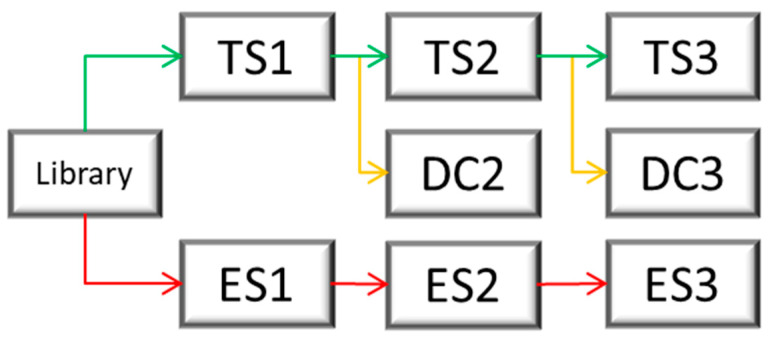
Schematic overview of the selection course. Two selections were started in parallel, the target selection 1 (TS1) and the empty selection 1 (ES1). All target selections had the D-tau(271-316) immobilized on the plate surface during selection (green arrow). All empty selections were devoid of the D-tau(271-316) immobilized on the plate surface during selection and were used as negative controls (red arrow). Direct controls (DC2 and DC3) were used as another form of negative control (yellow arrow). Enriched phages of the last target selection round were put onto plates where no D-tau(271-316) had been immobilized and a normal selection round was performed. Negative control sequences were compared with target selection sequences after NGS sequencing to value which peptides were not target-specific.

During the phage display selection experiment, multiple control selections were performed to ensure the quality of the selection. From the library, two starting selections were performed. The first, called TS1 for target selection 1, used a surface that contained the target, while the second one, called ES1 for empty selection 1, did not contain the target on the surface and served as a control. The empty selection was performed in parallel to the target selection. The phages derived from TS1 were later applied to the next target selection (TS2) as well as the direct control (DC2), which did not contain the target protein on the surface and served as a negative control for direct comparison. A subsequent third selection round was performed analogously. Figure 14 illustrates this course of the selection and at which points a control selection had been performed, which subsequently enabled the identification of the target-specific binders via the NGS results. The different selection types were named “target selection” (TS), “direct control” (DC), and “empty selection” (ES).

#### 4.4.2. Implementation

For the target selection rounds, 120 pmol of D-tau(271-316) was immobilized on a Pierce streptavidin plate (ThermoFisher Scientific, Waltham, MA, USA) via biotin-streptavidin binding. The target was solved in phosphate-buffered saline (PBS) buffer, and 100 µL was applied to each used well. For the empty selection and direct control selection, a buffer instead of D-tau(271-316) was used during immobilization. The subsequent procedure was identical for all three selections. The plate was incubated at room temperature for 60 min with shaking. After removing the immobilization solution, a blocking step with PBS and 10 mg/mL milk powder was performed. Two hundred microliters of the blocking buffer was incubated for 30 min under shaking at room temperature. The wells were washed six times with 200 µL washing buffer containing PBS with 2 mg/mL milk powder. The phages, with a total number of 2 × 10^11^, from a TriCo-16 phage display peptide library (Creative Biolabs, lot number: CBLX090318, Shirley, NY, USA), were solved in 100 µL PBS and added to the prepared wells. The incubation was performed at room temperature for 20 min with shaking. This was followed by 20 washing steps, each with 100 µL washing buffer. The elution of bound phages was performed by incubating the wells with 100 µL 0.2 M glycine HCl (pH 2.2) for 10 min at room temperature with shaking. The eluted phages were transferred into a prepared Eppendorf tube with 25 µL 1M tris-HCl (pH 9.1). Five microliters of the phages was used for output titer determination, and the rest of the phages were amplified in bacteria.

For phage amplification, 20 mL of *E.coli* K12 ER 2738 in lysogeny broth (LB) medium was added to a 250 mL flask and grown to an OD_600_ of 0.1. Tetracycline was added to an end concentration of 50 µM. The phage amplification was performed at 37 °C with shaking for 4.5 to 5 h. The 20 mL was harvested into 50 mL falcons and centrifuged at 3000× *g* at 4 °C for 20 min. The supernatant was mixed with 7 mL of PEG/NaCl (20% (*w*/*v*) Polyethylenglycol-8000, 2.5 M NaCl) and incubated overnight on ice. Afterwards, the solution was again centrifuged at 3000× *g* at 4 °C for 60 min. The supernatant was discarded and the pellet resuspended in 1 mL tris-buffered saline (TBS) and centrifuged for 5 min at 11,000× *g*. The supernatant was transferred into a new 1.5 mL Eppendorf tube. Two hundred microliters of PEG was added to the Eppendorf tube and incubated on ice for 60 min. After incubation, the mixture was centrifuged for 45 min at 4 °C at 3000× *g*. Finally, the resulting pellet containing the phages was properly resuspended in 100 µL TBS.

#### 4.4.3. Titer Determination

The output and input titer of each selection round were determined by diluting 1 µL of the output/input samples, taken at the end of each selection round and after amplification, and diluting it in 99 µL LB medium. Five and ten additional 1:10 dilutions were prepared for the output and input titer, respectively. One hundred microliters of *E. coli K12 ER2738* with an OD_600_ of 0.6 was added to each dilution. Eight hundred microliters of warm TOP-Agar were mixed with the 200 µL phage/bacteria mixture and put onto a small LB/IPTG/X-Gal plate. The plates were shaken in a double orbital manner to ensure sufficient distribution of the mixture throughout the plate. After brief drying at room temperature, the plates were flipped and incubated at 37 °C overnight. The respective output or input titer was determined by counting the resulting blue plaques on the plates with different dilution factors.

### 4.5. ELISA

For the ELISA, a Pierce Streptavidin/Thermo Scientific/15500 plate was used. D-tau(271-316) amounts of 120 pmol, 60 pmol, and 20 pmol were immobilized on the surface of the plate via biotin-streptavidin binding. The immobilization was performed in 100 µL PBS for 60 min at room temperature while shaking. The plate was blocked by incubation with 200 µL PBS containing 10 mg/mL milk powder for 60 min at room temperature and shaking. The wells were washed three times with 200 µL PBS containing 2 mg/mL milk powder and 0.1% Tween20. During the phage incubation, 5 × 10^10^ phages were used. The binding was performed in 100 µL PBS for 20 min at room temperature with shaking. An additional washing step with five repetitions and a washing volume of 100 µL was performed. The horseradish conjugated anti-M13 antibody from Sino Biological (Eschborn, Germany, product number 11973-MM05T-H) was diluted 1:2500, and 100 µL was incubated for 60 min at room temperature. Afterwards, each well was washed six times. One hundred microliters of 3,3′5,5′-tetramethylbenzidine (TMB) substrate solution was added to each well. After 30 s, the reaction was stopped by the addition of 100 µL 2 M sulfuric acid. The signal intensity was then quantified by detecting the absorption at 450 nm in a CLARIOstar microplate reader (BMG, Offenburg, Germany).

### 4.6. DNA isolation

For DNA isolation, 10 µL of purified phage suspension was diluted in 580 µL PBS 7.4. Two hundred microliters of PEG/NaCl was added, inverted eight times, and incubated at room temperature for 20 min. After incubation, the phage suspension was centrifuged at 21,000× *g* at 4 °C for 10 min. The supernatant was discarded, and the remaining pellet was resuspended in 200 µL of a 10:1 mixture of 3 M sodium acetate (pH 5.2) and TE-buffer (10 mM tris-HCl, pH 8, 0.1 mM EDTA). Afterwards, 500 µL of 99% ethanol was added and incubated for 15 min at room temperature, followed by 10 min centrifugation at 21,000× *g* at 4 °C. The supernatant was discarded, and the pellet was resuspended in 250 µL 70% ethanol. After a final centrifugation of 10 min at 21,000× *g* at 4 °C the supernatant was discarded, and the resulting pellet was dried in an Eppendorf concentrator 5301 (Eppendorf, Hamburg, Germany). The dried pellet was resuspended in 40 µL 10 mM tris buffer pH 8.

### 4.7. PCR

The DNA concentration was determined by photometric analysis using a nano photometer (IMPLEN, München, Germany). The PCR was performed with 25 ng of sample (2.5 µL), 0.2 µg of forward primer (5′-TCGTCGGCAGCGTCAGATGTGTATAAGAGACAGCGCAATTCCTTTAGTGGTACC-3′, 5 µL), 0.2 µg of reverse primer (5′-GTCTCGTGGGCTCGGAGATGTGTATAAGAGACAGCCCTCATAGTTAGCGTAACG-3′, 5 µL), and 12.5 µL of KAPA mix (Kapa Biosystems, Wilmington, USA) for a total volume of 25 µL. The PCR started at 3 min at 95 °C, followed by 25 cycles of 30 s at 95 °C, 30 s at 55 °C, and 30 s at 72 °C. The PCR finished at 5 min at 72 °C and was afterwards on hold at 4 °C. The success of PCR was verified by analysis with a DNA gel.

### 4.8. Next-Generation Sequencing

Next-generation sequencing (NGS) of the phage DNA was performed by the Genomics & Transcriptomics Labor of the Heinrich-Heine-Universität Düsseldorf. For the NGS, all the target selection inputs, as well as direct control input three and empty selection input three, were prepared and sequenced; this included a sample of the library.

### 4.9. Data Analysis

For the initial analysis and the grouping of the NGS data, the program TSAT (Target Sequence Analysis Tool) was used, which was developed in-house. A secondary analysis was performed using the clustering software Hammock.

#### 4.9.1. Target Sequence Analysis Tool (TSAT)

The program TSAT takes raw DNA data in a FASTA or FASTQ format and examines these data for the provided framing regions of the randomized peptide region. If the framing regions are present in the given DNA, the randomized region coding for the peptide is excised, grouped, translated into amino acids, and saved in a database. Once saved in a database, the data are further processed by filtering. During filtering, the sequence frequency of the last target selection round are analyzed for their enrichment from target round to target round as well as compared to the empty selection and direct control. The filtering is conducted according to the following formula:

TS3≥ES3 & TS3≥DC3 & TS3≥TS2 & TS2≥ES2 & TS2≥DC2 & TS2≥TS1 & TS1≥ES1& TS1≥Library

Only sequence abundancies or frequencies which pass the filter and possess more than eight amino acid residues are added to the new database. Each sequence in the new database is assigned an enrichment score and an empty score. These are based on the ratio between the last target selection round, the library, and the last empty selection round. The scores are calculated according to the following formulas:

$$\mathrm{Enrichment}\;\mathrm{score}=\frac{\mathrm{frequency}\;\mathrm{of}\;a\;\mathrm{sequence}\;\mathrm{in}\;\mathrm{TS}3}{\mathrm{frequency}\;\mathrm{of}\;\mathrm{the}\;\mathrm{same}\;\mathrm{sequence}\;\mathrm{in}\;\mathrm{library}}$$

$$\mathrm{Empty}\;\mathrm{score}=\frac{\mathrm{frequency}\;\mathrm{of}\;a\;\mathrm{sequence}\;\mathrm{in}\;\mathrm{TS}3}{\mathrm{frequency}\;\mathrm{of}\;\mathrm{the}\;\mathrm{same}\;\mathrm{sequence}\;\mathrm{in}\;\mathrm{ES}3}$$

If a sequence was not found in the library or the last empty selection, then the lowest value of the corresponding selection is taken and divided by two. Afterwards, this value is taken to calculate the enrichment or empty score. This was decided to first solve the problem of dividing by zero in the cases where the sequence was not found in the empty selection or library and second to highlight these sequences as they either enriched themselves strongly or were very target-specific. All calculations are performed as parts per million (ppm), which means the abundance of the particular sequence within the “read” divided by the total number of sequences obtained by the “read” and multiplied by 1 million, to enable a comparison between the samples. Finally, a document is created where all the sequences that passed the filter are displayed in a descending order according to their empty score and in FASTA format. This final document can be used to run the Hammock software.

#### 4.9.2. Hammock

The program Hammock is software intended for the sequence clustering of large datasets comprising short sequences [21]. It was used to generate clusters of similar sequences to generate motifs with a high order of similarity. Hammock was performed using the standard parameters of the software.

#### 4.9.3. Peptides TF2D-5, TF2D-5a, and CP1

All the peptides used in this study were ordered from CASLO (Lyngby, Denmark). Both TF2D-5 and CP1 are 16-mers, while TF2D-5a is a 21-mer. The respective sequences are:
TF2D-5dGGyqilfkipGGhihTF2D-5adGGyqilfkipGGhihrrrrrCP1GiiGpqykdhGhfliG

All the peptides were synthesized from D-enantiomeric amino acid building blocks (except glycine) with the C-terminus amidated. TF2D-5 was selected during mirror-image phage display selection, while CP1 is a control peptide with the same amino acid residues as in TF2D-5, but in a randomized sequence. TF2D-5a was designed to increase the ability of TF2D-5 to cross membranes.

### 4.10. Recombinant Full-Length Tau Expression and Purification

The gene for the human full-length tau was prepared on a plasmid called pET28a(+)-tau2N4R. *E. coli* pRare cells were transformed using the generated plasmid. This was achieved by adding 1 µL (approx. 100 ng) of the plasmid DNA to 100 µL of competent E.coli pRare cells. The mix was incubated for 30 min on ice, followed by heat shock treatment at 42 °C for 45 s. After incubation on ice for 5 min, 900 µL of LB media was added and incubated for 1 h at 37 °C and 130 rpm. Transformation efficiency was controlled by plating out 100 and 800 µL of cell solution onto agar plates containing kanamycin and chloramphenicol. The remaining 100 µL was used to inoculate 100 mL of LBKAN, CHL medium and incubated overnight at 37 °C with shaking at 130 rpm.

For full-length tau expression, a preculture of transformed E.coli pRare cells was grown overnight in LBKAN, CHL at 37 °C with 5% CO2 and shaking at 300 rpm. The antibiotic concentration was adjusted to 1 mM. The overnight culture was transferred to the expression culture and adjusted to an OD600 of 0.1. LBKAN, CHL medium was used as the expression medium. The culture was grown to an OD600 of 0.6 0.7 and expression was induced by adding 1 mM IPTG. Expression was performed at 37 °C at 300 rpm, with shaking for 3 to 4 h. Cells were harvested by centrifugation at 5000× *g* for 20 min at 4 °C. The resulting pellets were either stored at −20 °C or resuspended directly in lysis buffer containing 500 mM NaCl, 20 mM PIPES, 1 mM EDTA, and 2 mM 2-mercaptoethanol pH 7.0.

The resuspended cell pellets were boiled for 15–20 min to disrupt all the cells and denature most of the proteins. As tau is an intrinsically disordered protein, the target protein is not destroyed by this method. Full-length tau is separated from the denatured proteins and cell debris by centrifugation for 1 h at 11,000× *g* at 4 °C. In preparation for ion exchange chromatography, the supernatant was dialyzed against 20 mM PIPES, 50 mM NaCl, 2 mM DTT, 0.5 mM EDTA; pH 6.7. Two dialysis steps were performed, with the first at room temperature for one hour and the second overnight at 4 °C. The next day, cation exchange chromatography was performed using a Bio SCX, NP5, SS column from Agilent Technologies (Santa Clara, CA, USA) on a 1200 series HPLC from Agilent Technologies. The elution buffer used was 20 mM PIPES 1 M NaCl, 2 mM DTT, and 0.5 mM EDTA; pH 6.7. The collected fractions were pooled and dialyzed overnight against 20 mM PIPES, 150 mM NaCl, 2 mM DTT; pH 7.4. A size exclusion run on a 1200 series HPLC from Agilent Technologies was performed. For the size exclusion, a Bio SEC 3, 300Å column form Agilent Technologies was used. The concentration of purified tau was measured and afterwards stored at −80 °C.

### 4.11. Screening for Aggregation Inhibitors via Thioflavin-T Assay

To identify the peptides (compounds) with the highest chance of becoming a successful therapeutic agent, a screening for aggregation inhibitors via ThT assay was performed. All the compounds were only tested in one concentration and added at the start of the experiment. The specifics of the other shown ThT assays are described in the next part. A FLUOstar plate reader (BMG, Offenburg, Germany) was used for the experiment. Fifty micrometers of recombinant full-length tau 441 was mixed with 12.5 µM heparin, 2% DMSO, 20 µM ThT, 500 µM DTT, and 50 µM compound. The total volume was adjusted to 100 µL with PBS pH 7.4, and the plate (Corning, New York, NY, USA) was measured with bottom optics. The experiment ran at 37 °C with measurement intervals of 20 min. Before each measurement, the plate was shaken for 15 s at 300 rpm.

### 4.12. Thioflavin-T Assay

ThT assays to characterize the aggregation inhibition properties of the selected compounds were conducted as follows. All measurements were performed in a FLUOstar plate reader (BMG, Offenburg, Germany). Recombinant full-length tau 441 (25 µM) was mixed with heparin (12.5 µM), DTT (200 µM), NaN_3_ (0.05%), and ThT (20 µM). Slight differences in tau concentrations compared to former THT assays are justified by different tau preparations which exhibit different aggregation properties. The compounds were added with a maximum concentration of 25 µM. The total volume was adjusted to 100 µL with PBS pH 7.4, and the plate (Corning, New York, NY, USA) was measured with bottom optics. A 3 mm borosilicate bead (Hilgenberg GmbH, Malsfeld, Germany) was added to each well. The experiment ran at 37 °C, with measurement intervals of 20 min. Before each measurement, the plate was shaken for 15 s at 300 rpm.

### 4.13. Atomic Force Microscopy (AFM)

All AFM measurements were performed by drying 1 µL of sample on mica followed by two washing steps using 100 µL water. All samples were measured on a JPK Nanowizard3 with an intermittent contact mode on air and an OMCL AC160TS as a cantilever.

### 4.14. SPR Measurement

#### 4.14.1. Chip Preparation

The chips were prepared using a Biacore T200 (Biacore, GE Healthcare, Uppsala, Sweden) via manual run as well as a Biacore 8K (Biacore, GE Healthcare, Uppsala, Sweden) using an immobilization run. Series S CM5 chips were used, and the flow rate was adjusted to 10 µL/min. A mixture of 50 mM N-hydroxysuccinimide (NHS) and 16.1 mM *N*-Ethyl-*N*′-(dimethylaminopropyl)carbodiimide (EDC) (XanTec, Düsseldorf, Germany) was used to activate the chip. The activation duration was 7 min with a flow rate of 10 µL/min. Prior to immobilization, TF2D-5 and TF2D-5a were diluted with 10 mM sodium acetate pH 5 to a concentration of 50 µg/mL and 5 µg/mL, respectively. Each compound was immobilized on separate chips, with the total immobilization level being 130 RU for TF2D-5 and 600 RU for TF2D-5a. Channel one of each chip was used as the reference and only buffer was injected during immobilization for the T200. Flow cell 1 was used as a reference during experiments on the 8K. Thee quenching of both channels (T200) and flow cells (8K) was achieved by injection of 1 M ethanolamine, pH 8.5 (XanTec, Düsseldorf, Germany) for 7 min.

#### 4.14.2. Measurement T200

Measurements of TF2D-5 and TF2D-5a against full-length tau 441 were performed on a Biacore T200 (Biacore, GE Healthcare, Uppsala, Sweden) using multicycle experiments. The flow rate was adjusted to 35 µL/min. Full-length tau 441 with different concentrations was used as an analyte. TBS pH 7.4 with 0.05% Tween20 and 10 µM DTT was used as a running buffer. Before measurement, three startup cycles without analyte were performed to allow for instrument equilibration. The experiment started with two cycles of 0 nM analyte concentration, used to reference the background noise within the experiment. The tau association time was set to ten min and the dissociation time to 60 min. The tau concentrations were 3.1 nM, 6.2 nM, 12.5 nM, and 25 nM for TF2D-5 and 31.2 nM, 62.5 nM, 125 nM, 250 nM, and 500 nM for TF2D-5a. The remaining tau was eluted by three 45 s regeneration cycles with 2 M guanidinium hydrochloride. The data were evaluated using the Biacore T200 Evaluation Software 3.2 using a 1:1 fit (RI = 0).

#### 4.14.3. Measurement 8K

Measurements of TF2D-5a against the R3 domain of tau were performed on a Biacore 8K (GE Healthcare, Uppsala, Sweden) using a single cycle experiment. The all L-enantiomeric R3 domain (^304^SVQIVYKPVDLSKVTSKCGSLGNIHHKPGGG^336^) of tau, purchased from CASLO (CASLO ApS, Lyngby, Denmark), was used with concentrations of 3.1 nM, 6.2 nM, 12.5 nM, 25 nM, 50 nM, and 100 nM as the analytes. PBS pH 7.4 with 0.05% Tween20 was used as a running buffer. The flow rate was adjusted to 35 µL/min. Before measurement, three startup cycles without analyte were performed to allow for instrument equilibration. The contact time for each concentration was 3 min. The final dissociation time was set to 60 min. The data were analyzed using the Biacore insight evaluation software 3.0.12.15655 using a 1:1 fit.

### 4.15. MST Measurements

The affinity of TF2D-5 and TF2D-5a for full-length tau was additionally tested by MST measurements. All peptides were labeled with the fluorophore CF633 kit obtained from Sigma Aldrich (USA) and purified by reverse phase HPLC.

#### 4.15.1. TF2D-5

CF633-labeled TF2D-5 was used at a concentration of 500 nM, and the tau concentration ranged from 2.5 µM to 0.2 pM using a dilution series of 1:2. The full-length tau was centrifuged at 20,000× *g* at 4 °C for 20 min, after thawing before use. The measurement was performed in 1x PBS pH 7.4 containing 150 mM NaCl.

#### 4.15.2. TF2D-5a

CF633-labeled TF2D-5a was used at a concentration of 62.5 nM, and the tau concentration ranged from 300 nM to 0.5 nM using a 2:1 dilution series. Full-length tau was centrifuged at 20,000× *g* at 4 °C for 20 min, after thawing before use. Measurements were performed in a 0.1 M sodium phosphate buffer of pH 7.4 containing 75 mM sodium sulfate and 0.05% Tween 20. All measurements were repeated four times. The average of all the measurements was calculated, and a sigmoidal fit (Boltzmann) was used for KD determination.

#### 4.15.3. CP1

CF633-labeled CP1 was used at a concentration of 62.5 nM, and the tau concentration ranged from 2.5 µM to 0.2 pM using a dilution series of 1:2. Full-length tau was centrifuged at 20,000× *g* at 4 °C for 20 min, after thawing before use. Measurements were performed in 0.1 M sodium phosphate buffer of pH 7.4 containing 75 mM sodium sulfate and 0.05% Tween 20. All measurements were repeated four times. The average of all the measurements was calculated.

### 4.16. Creation of Soluble Full-Length Tau Aggregates

First, the insoluble PFF were produced by the incubation of 130 µM recombinant tau in a LoBind reaction tube (Eppendorf GmbH, GE) with one borosilicate glass bead (d = 3.0 mm; Hilgenberg, DE) in 1× PBS pH 7.4 with 65 µM Heparin, 1 mM DTT, and 0.05% (*w*/*v*) sodium azide for two weeks at 37 °C. The insoluble PFF were harvested by ultracentrifugation at 100.000× *g* for 30 min at 4 °C, and the pellet was washed several times with 1× PBS pH 7.4. The monomer equivalent concentration was determined by measuring the tau concentration in the supernatant after the first centrifugation and subtracting it from the starting concentration for fibrilization. The insoluble PFF were resuspended in buffer and frozen at −80 °C with liquid N2. PFF oligomers were generated by harsh sonication of 200 µL insoluble Tau with a 13 µM monomer equivalent concentration for 3 × 15 s (1 s. on/off) and 60% amplitude with a tip sonifier (MS 72 micro tip, Sonopolus, Brandelin, GE). The insoluble PFF were separated by centrifugation at 100.000× *g* for 1 h at 4 °C. The supernatant containing the PFF oligomers was separated, aliquoted, and frozen at −80 °C with liquid N2.

### 4.17. Monomer Quantification Using HPLC

Quantities equaling 100 nM soluble tau oligomers and 100 nM of monomeric tau were incubated for 96 h at 37 °C at 300 rpm. Sub-stoichiometric concentrations of TF2D-5, TF2D-5a, and CP1 (25 µM, 50 µM) were added. After 96 h, the samples were centrifuged for two min at 11,000× *g* to sediment the large aggregates. One hundred microliters of each sample was analyzed on an HPLC (Agilent 1200 with quaternary pump, manual injection valve, and variable wavelength detector) with a size exclusion column (Agilent Bio-SEC 3, 300 A, 7.8 × 300 mm, 3 µm). The flow rate was adjusted to 1 mL/min.

### 4.18. Stability Assay of D-Peptides in Simulated Human Body Fluids

The stability of the compounds was tested according to Elfgen et al. [33] and Schemmert et al. [34] Exceptions included the extraction of plasma, simulated gastric fluid (SGF), and simulated intestinal fluid (SIF) samples. These were obtained by the addition of 12.5 µL 0.35 M ZnSO_4_, followed by vortexing, the addition of 12.5 µL 0.5 M HCl, additional vortexing, and centrifugation at 14,000× *g* for 10 min at 4 °C.

### 4.19. Cell-Viability Assay

Human bone marrow SH-SY5Y cells and rat pheochromocytoma PC12 cells were cultivated in RPMI medium with 10% fetal bovine serum and 5% horse serum. On a collagen-coated 96-well plate (Gibco, Life Technology, Carlsbad, CA, USA), 10.000 cells were seeded per well. The plate was incubated for 24 h at 37 °C and 5% CO_2_. TF2D-5 and TF2D-5a were added to the cells at concentrations of 0.2 µM, 1 µM, and 5 µM, respectively. As a positive control, 0.1% Trion X-100 was used. A medium was used as a negative control. The samples were incubated for a further 24 h at 37 °C and 5% CO_2_. Cell viability was determined by using the Cell Proliferation Kit 1 (MTT) (Roche, Basel, Switzerland) according to the instruction manual. The absorbance of generated formazan products was measured using a CLARIOstar plate reader (BMG, Offenburg, Germany). Absorption at 570 nm was measured and referenced against absorbance at 660 nm.

### 4.20. Blood–Brain Barrier (BBB) Permeability Assay

The preparation of the blood–brain barrier (BBB) model was performed according to Liu et al. [35], Niego et al. [36], and Pardridge et al. [37]. Brain endothelial cells from rats and astroglial cells were used to cultivate the different sides of the porous membrane.

Brain endothelial cells from rats (RBMVEC, Cell Applications Inc., R840-05a, San Diego, CA, USA) were cultivated in Complete Classic medium (Cell Systems, Kirkland, WA, USA, 4Z0-500), and rat astroglial cells (CTX TNA2, ATCC, CRL-2006) were cultivated in Complete Classic medium (Cell Systems, Kirkland, WA, USA, 4Z0-500). The cells were allowed to grow in a humidified incubator with 5% CO_2_ at 37 °C and a maximum of 20 passages. The cells were passaged every 3 to 5 days, according to their confluence.

In order to set up the in vitro BBB model, the astroglial cells were seeded at a density of 600.000 cells/cm² on the outer (basolateral, “brain”) side of an insert with a porous membrane (Corning Transwell polycarbonate membrane cell culture inserts, CLS3415, 3 µM pore size) and allowed to grow/adhere overnight in the Complete Classic medium. The next day, brain endothelial cells were seeded at a density of 600.000 cells/cm² on the inner side (apical, “blood”) of the corresponding membrane. The seeded inserts were then cultivated to grow to full confluence over a period of 5 to 7 days, with the medium renewed daily. On the day of the assay, the medium was swapped out for Complete Serum-Free medium (Cell Systems, SF-4Z0-500), and the D-peptides TF2D-5 and TF2D-5a were added at a final concentration of 1 mg/mL to the inner chamber. Prior to this, TEER (transendothelial electrical resistance values) were measured in each well to ensure the integrity of the in vitro BBB. At 0, 30, 60, 90, 120, 150, 180, and 240 min, 50 µL of the medium out of the outer chamber was taken and analyzed by HPLC. This 50 µL would then be replaced by the same amount of fresh serum-free medium to keep the fluid levels equal across both chambers. This slight dilution was factored into the following calculations. Furthermore, a dilution series of each reagent was analyzed alongside the samples as a calibration to quantify the analysis. Each substance and time point was tested in triplicate plus a negative control, in which the same set-up was used without cells. The P_app_ values were calculated as follows (higher is better):

$$\mathrm{Papp}\;=\left(\frac{V}{A}*C\right)*\left(\mathrm{Dc}/\mathrm{Dt}\right)$$

V = Volume of basolateral chamber (mL);

A = Membrane area (cm2);

C = Concentration (µg/µL);

Dc = Measured concentration in basolateral chamber (µg/mL);

Dt = sampling time (s).

### 4.21. Gel Electrophoresis

Each replicate of the screening via ThT assay was collected and pooled. The samples were centrifuged at 4 °C at 11,000× *g* for 60 min. The supernatant and pellet were subsequently separated, and the pellet was resuspended in the same volume. The samples were mixed 1:4 with 4× Lämmli and heated to 95 °C. Afterwards, the samples were loaded onto a 15% gel and run at 45 mA per gel for 45 min. Colorization of the gels was achieved by incubation with Coomassie brilliant blue for 30 min. The gels were afterwards decolored by heating them in water with a microwave and subsequent incubation in the heated water with paper tissues. The gels were imaged subsequently on a Gel Doc XR+ Gel Documentation System (Bio-Rad Laboratories, Hercules, CA, USA).

### 4.22. Cell Assay for Tau Aggregation

A construct encoding the four microtubule binding repeats of the human microtubule-associated protein tau (amino acids 243 to 375, corresponding to 2N4R tau), with the familial mutations P301L and V337M was fused via an 18-amino-acid flexible linker (EFCSRRYRGPGIHRSPTA) to YFP at the C-terminus of tau and introduced into the pMK–RQ expression vector (GeneArt; Thermo Fisher Scientific, Waltham, MA, USA). The tauK18(LM)-YFP construct was subcloned into the pIRESpuro3 vector (Clontech; Takara Bio, JPN) using EcoRI (5′) and NotI (3′) restriction sites. HEK293T cells (American Type Culture Collection) were cultured in high-glucose Dulbecco’s Modified Eagle’s Medium (DMEM; Sigma-Aldrich, St. Louis, MI, USA), supplemented with 10% (*v*/*v*) fetal calf serum (Sigma-Aldrich, USA) and 50 units/mL penicillin, as well as 50 μg/mL streptomycin (Sigma-Aldrich, USA). The cells were cultured in a humidified atmosphere of 5% CO_2_ at 37 °C. The cells plated in DMEM were transfected using lipofectamine 2000 (Invitrogen; Thermo Fisher Scientific, USA). The stable cells were selected in DMEM containing 1 μg/mL puromycin (EMD Millipore, Burlington, MA, USA). Monoclonal lines were generated by fluorescence-activated cell sorting of a polyclonal cell population in 96-well plates using a MoFlo XDP cell sorter (Beckman Coulter, USA). Finally, the clonal cell line H7 was selected from among 16 clonal cell lines and is referred to as tauK18(LM)–YFP cells. The compounds were incubated with 1.5% (*v*/*v*) lipofectamine 2000 in OptiMEM for 2 h at room temperature. The tauK18(LM)–YFP cells were plated in a 384-well plate with poly-D-lysine coating (Greiner, AT) at a density of 1.000 cells per well with 0.1 μg/mL Hoechst 33342 (Thermo Fisher Scientific, USA), and the previously prepared transfection mix was added directly to the cells in the well. To seed the cellular aggregation of tau in the tauK18(LM)–YFP cells, 25 nM of sonicated tau fibrils (seeds) was incubated with 1.5% (*v/v*) lipofectamine in OptiMEM for 2 h at room temperature and added to each well 3 h after the first transfection. The plate was then incubated in a humidified atmosphere of 5% CO_2_ at 37 °C. On day 3, the cells were imaged with an IN Cell Analyzer 6500HS System (Cytiva, SE) using the blue and green fluorescence channels and analyzed using IN Carta Image Analysis Software (Cytiva, SE) after an algorithm was established to identify intracellular aggregates in the living cells. Statistical analysis was performed using one-way ANOVA, followed by Dunnett’s multiple comparisons test (GraphPad Prism 9, GraphPad Software, USA).

## 5. Conclusions

In this report, we showed the successful selection of the all D-enantiomeric compounds TF2D-5 and TF2D-5a. Both compounds bind the monomeric form of the tau protein and are able to significantly delay its aggregation. We showed that both compounds have a high affinity towards monomeric tau and are mostly stable in different simulated body fluids. Positive results using the blood–brain barrier model suggest that both compounds should be able to cross the said barrier, while the cell culture model showed an effective inhibition of tau aggregation by the compounds in a cellular environment. Summarizing these results, we believe that TF2D-5 and TF2D-5a are promising therapeutic candidates for Alzheimer’s disease and other tauopathies.

## Data Availability

Not applicable.

## Supplementary Materials

The supporting information can be downloaded at: https://www.mdpi.com/article/10.3390/ijms24032161/s1.

## References

[1] McKhann G., Drachman D., Folstein M., Katzman R., Price D., Stadlan E.M. (1984). Clinical diagnosis of Alzheimer‘s disease: Report of the NINCDS-ADRDA Work Group under the auspices of Department of Health and Human Services Task Force on Alzheimer‘s Disease. Neurology.

[2] Fiock K.L., Smalley M.E., Crary J.F., Pasca A.M., Hefti M.M. (2020). Increased Tau Expression Correlates with Neuronal Maturation in the Developing Human Cerebral Cortex. Eneuro.

[3] Ikegami S., Harada A., Hirokawa N. (2000). Muscle weakness, hyperactivity, and impairment in fear conditioning in tau-deficient mice. Neurosci. Lett..

[4] Drubin D.G., Feinstein S.C., Shooter E.M., Kirschner M.W. (1985). Nerve growth factor-induced neurite outgrowth in PC12 cells involves the coordinate induction of microtubule assembly and assembly-promoting factors. J. Cell Biol..

[5] Kirschner D.A., Inouye H., Duffy L.K., Sinclair A., Lind M., Selkoe D.J. (1987). Synthetic peptide homologous to beta protein from Alzheimer disease forms amyloid-like fibrils in vitro. Proc. Natl. Acad. Sci. USA.

[6] Wischik C.M., Novak M., Thogersen H.C., Edwards P.C., Runswick M.J., Jakes R., Walker J.E., Milstein C., Roth M., Klug A. (1988). Isolation of a fragment of tau derived from the core of the paired helical filament of Alzheimer disease. Proc. Natl. Acad. Sci. USA.

[7] Kent S.A., Spires-Jones T.L., Durrant C.S. (2020). The physiological roles of tau and Abeta: Implications for Alzheimer’s disease pathology and therapeutics. Acta Neuropathol..

[8] Elmaleh D.R., Farlow M.R., Conti P.S., Tompkins R.G., Kundakovic L., Tanzi R.E. (2019). Developing Effective Alzheimer’s Disease Therapies: Clinical Experience and Future Directions. J. Alzheimer’s Dis..

[9] Reas E.T. (2017). Amyloid and Tau Pathology in Normal Cognitive Aging. J. Neurosci..

[10] Huber C.M., Yee C., May T., Dhanala A., Mitchell C.S. (2017). Cognitive Decline in Preclinical Alzheimer’s Disease: Amyloid-Beta versus Tauopathy. J. Alzheimer’s Dis..

[11] Hooper C., Killick R., Lovestone S. (2008). The GSK3 hypothesis of Alzheimer’s disease. J. Neurochem..

[12] Von Bergen M., Friedhoff P., Biernat J., Heberle J., Mandelkow E.M., Mandelkow E. (2000). Assembly of tau protein into Alzheimer paired helical filaments depends on a local sequence motif ((306)VQIVYK(311)) forming beta structure. Proc. Natl. Acad. Sci. USA.

[13] Von Bergen M., Barghorn S., Li L., Marx A., Biernat J., Mandelkow E.M., Mandelkow E. (2001). Mutations of tau protein in frontotemporal dementia promote aggregation of paired helical filaments by enhancing local beta-structure. J. Biol. Chem..

[14] Seidler P.M., Boyer D.R., Rodriguez J.A., Sawaya M.R., Cascio D., Murray K., Gonen T., Eisenberg D.S. (2017). Structure-based inhibitors of tau aggregation. Nat. Chem..

[15] Fitzpatrick A.W.P., Falcon B., He S., Murzin A.G., Murshudov G., Garringer H.J., Crowther R.A., Ghetti B., Goedert M., Scheres S.H.W. (2017). Cryo-EM structures of tau filaments from Alzheimer’s disease. Nature.

[16] Schumacher T.N.M., Mayr L.M., Minor D.L., Milhollen M.A., Burgess M.W., Kim P.S. (1996). Identification of d -Peptide Ligands Through Mirror-Image Phage Display. Science.

[17] Wiesehan K., Willbold D. (2003). Mirror-image Phage Display: Aiming at the Mirror. Chembiochem.

[18] Funke S.A., Willbold D. (2009). Mirror image phage display—A method to generate d-peptide ligands for use in diagnostic or therapeutical applications. Mol. Biosyst..

[19] Sun N., Funke S.A., Willbold D. (2012). Mirror image phage display—Generating stable therapeutically and diagnostically active peptides with biotechnological means. J. Biotechnol..

[20] Santur K., Reinartz E., Lien Y., Tusche M., Altendorf T., Sevenich M., Tamgüney G., Mohrlüder J., Willbold D. (2021). Ligand-Induced Stabilization of the Native Human Superoxide Dismutase 1. ACS Chem. Neurosci..

[21] Krejci A., Hupp T.R., Lexa M., Vojtesek B., Muller P. (2015). Hammock: A hidden Markov model-based peptide clustering algorithm to identify protein-interaction consensus motifs in large datasets. Bioinformatics.

[22] Futaki S., Goto S., Sugiura Y. (2003). Membrane permeability commonly shared among arginine-rich peptides. J. Mol. Recognit..

[23] Wen J., Hong L., Krainer G., Yao Q.-Q., Knowles T.P.J., Wu S., Perrett S. (2021). Conformational Expansion of Tau in Condensates Promotes Irreversible Aggregation. J. Am. Chem. Soc..

[24] Asha S., Vidyavathi M. (2009). Role of Human Liver Microsomes in In Vitro Metabolism of Drugs—A Review. Appl. Biochem. Biotechnol..

[25] Sanders D.W., Kaufman S.K., DeVos S.L., Sharma A.M., Mirbaha H., Li A., Barker S.J., Foley A.C., Thorpe J.R., Serpell L.C. (2014). Distinct Tau Prion Strains Propagate in Cells and Mice and Define Different Tauopathies. Neuron.

[26] Woerman A.L., Aoyagi A., Patel S., Kazmi S.A., Lobach I., Grinberg L.T., McKee A.C., Seeley W.W., Olson S.H., Prusiner S.B. (2016). Tau prions from Alzheimer’s disease and chronic traumatic encephalopathy patients propagate in cultured cells. Proc. Natl. Acad. Sci. USA.

[27] Aoyagi A., Condello C., Stohr J., Yue W., Rivera B.M., Lee J.C., Woerman A.L., Halliday G., van Duinen S., Ingelsson M. (2019). Abeta and tau prion-like activities decline with longevity in the Alzheimer’s disease human brain. Sci. Transl. Med..

[28] Van Groen T., Wiesehan K., Funke S.A., Kadish I., Nagel-Steger L., Willbold D. (2008). Reduction of Alzheimer‘s disease amyloid plaque load in transgenic mice by D3, A D-enantiomeric peptide identified by mirror image phage display. ChemMedChem.

[29] Malhis M., Kaniyappan S., Aillaud I., Chandupatla R.R., Ramirez L.M., Zweckstetter M., Horn A.H.C., Mandelkow E., Sticht H., Funke S.A. (2021). Potent Tau Aggregation Inhibitor D-Peptides Selected against Tau-Repeat 2 Using Mirror Image Phage Display. Chembiochem.

[30] Aillaud I., Kaniyappan S., Chandupatla R.R., Ramirez L.M., Alkhashrom S., Eichler J., Horn A.H.C., Zweckstetter M., Mandelkow E., Sticht H. (2022). A novel D-amino acid peptide with therapeutic potential (ISAD1) inhibits aggregation of neurotoxic disease-relevant mutant Tau and prevents Tau toxicity in vitro. Alzheimer’s Res. Ther..

[31] Huseby C., Bundschuh R., Kuret J. (2019). The role of annealing and fragmentation in human tau aggregation dynamics. J. Biol. Chem..

[32] Kutzsche J., Jürgens D., Willuweit A., Adermann K., Fuchs C., Simons S., Windisch M., Hümpel M., Rossberg W., Wolzt M. (2020). Safety and pharmacokinetics of the orally available antiprionic compound PRI-002: A single and multiple ascending dose phase I study. Alzheimer’s Dement. Transl. Res. Clin. Interv..

[33] Elfgen A., Hupert M., Bochinsky K., Tusche M., Gonzalez de San Roman Martin E., Gering I., Sacchi S., Pollegioni L., Huesgen P.F., Hartmann R. (2019). Metabolic resistance of the D-peptide RD2 developed for direct elimination of amyloid-beta oligomers. Sci. Rep..

[34] Schemmert S., Camargo L.C., Honold D., Gering I., Kutzsche J., Willuweit A., Willbold D. (2021). In Vitro and In Vivo Efficacies of the Linear and the Cyclic Version of an All-d-Enantiomeric Peptide Developed for the Treatment of Alzheimer‘s Disease. Int. J. Mol. Sci..

[35] Liu H., Funke S.A., Willbold D. (2010). Transport of Alzheimer disease amyloid-beta-binding D-amino acid peptides across an in vitro blood-brain barrier model. Rejuvenation Res..

[36] Niego B., Medcalf R.L. (2013). Improved method for the preparation of a human cell-based, contact model of the blood-brain barrier. J. Vis. Exp..

[37] Pardridge W.M., Triguero D., Yang J., Cancilla P.A. (1990). Comparison of in vitro and in vivo models of drug transcytosis through the blood-brain barrier. J. Pharmacol. Exp. Ther..

