# Supplementary material for: Stabilization of Monomeric Tau Protein by All D-Enantiomeric Peptide Ligands as Therapeutic Strategy for Alzheimer’s Disease and Other Tauopathies
Source: Int J Mol Sci. 2023 Jan 21;24(3):2161. doi: 10.3390/ijms24032161 (PMC9917023; doi:10.3390/ijms24032161)
Supplement: Supplementary file 1 [file ijms-24-02161-s001.zip › ijms-2086723-supplementary.pdf]

International Journal of
Molecular Sciences

MDPI

Supplementary material

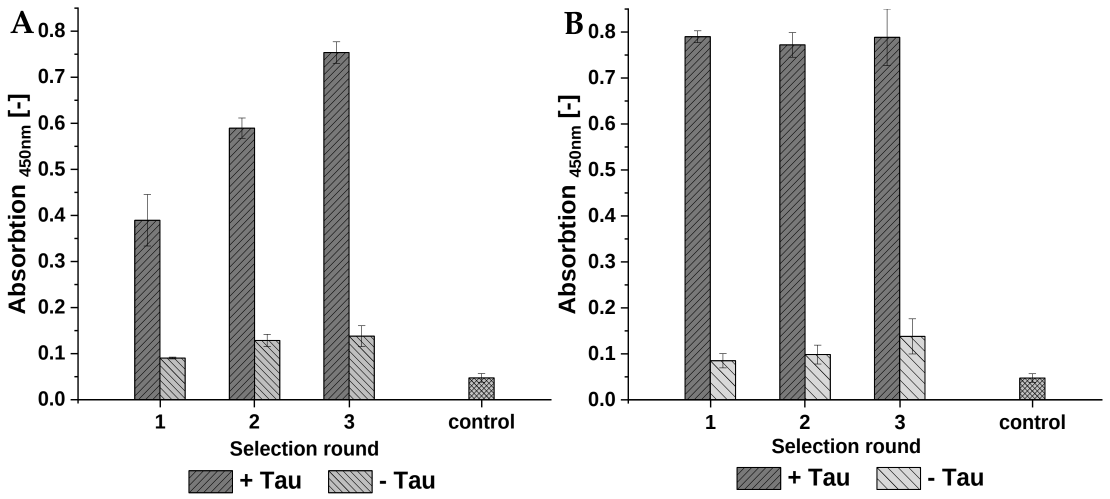

Figure S1. Enrichment ELISA with different concentration of immobilized D-tau(271-316). The plate for the enrichment ELISA was coated with 60 pmol **(A)** and 200 pmol **(B)**. D-tau(271-316), shown in dark grey, was incubated with 5*1010 phages. Buffer was added in-stead of the D-tau(271-316) during coating to act as a negative control (light grey). Lastly a separate control was performed to estimate the background noise level. This control did not contain any phages but was otherwise performed as the ELISA with D-tau(271-316). The signal intensity correlates with the amount of phages that are retained after several washing steps. We observed an increase of signal with higher selection rounds for wells that were coated with 60 pmol of D tau(271-316), while the signal for wells that were coated with buffer only showed a small increase. This indicates the en-richment of phages that are specific for the D-tau(271-316). Wells that were coated with 200 pmol of D tau(271-316) showed no increase of signal intensity with selection round but reached the maxi-mum ab-sorption of about 0.8 in the first selection round.

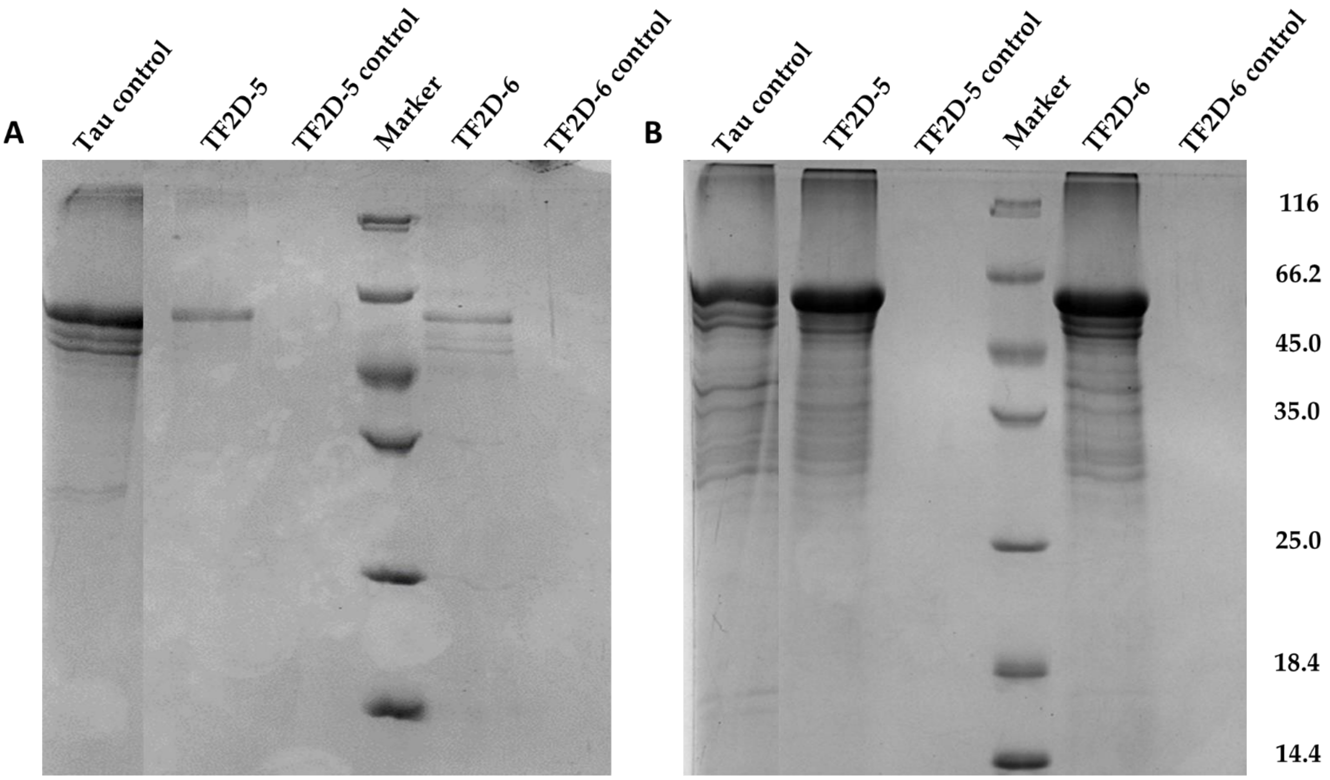

Figure S2. SDS-gel electrophoresis shows the impact of different selected compounds. Replicates from the screening ThT were collected pooled and centrifuged for 1 h at 11000 g and 4°C. Afterwards the supernatant and pellet were separated. The pellet was resuspended in the same volume as the supernatant and both were analyzed on different 15 % SDS gels. Both gels ran for 45 min with 45 mA. **A)** Depicted are the results of the SDS gel that ran the resuspended pellet. Smaller bands visible at the height that tau runs are present for TF2D-5 and TF2D-6. **B)** Depicted are the results for the SDS gel that ran the supernatant sample. Strong bands are visible at the height that tau runs are present for TF2D-5 and TF2D-6.

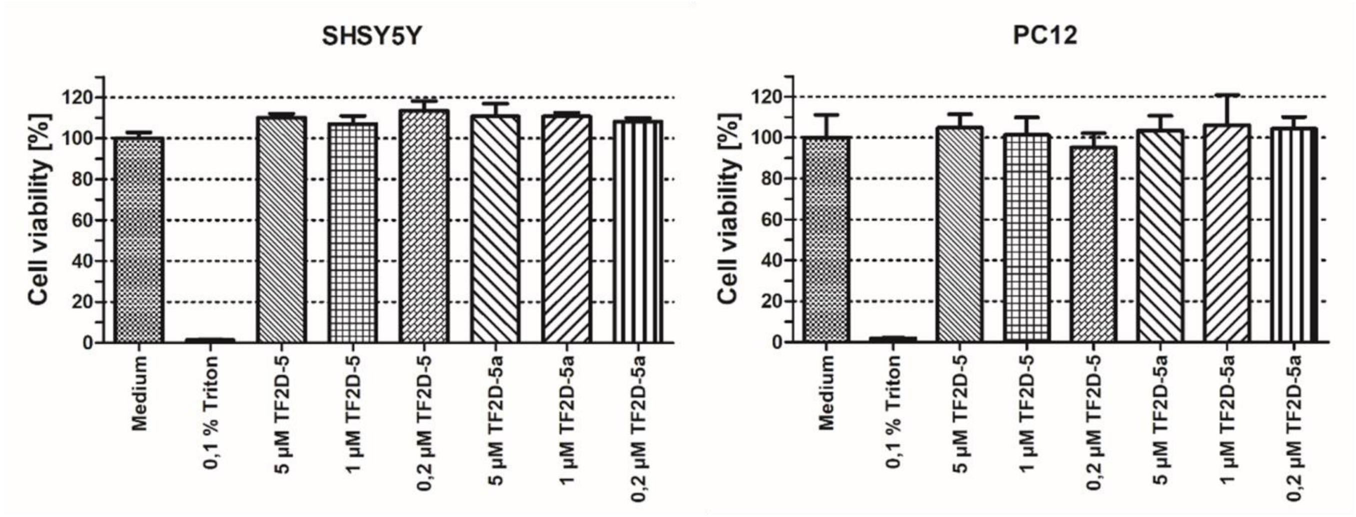

Figure S3. Peptide toxicity assay for different concentrations of TF2D-5 and TF2D-5a. For this assay the compounds TF2D-5 and TF2D-5a were incubated on SHSY5Y and PC12 cells for 24 h at 37 °C and 5 % CO2. The "Cell Proliferation Kit 1" from Roche was used for the MTT assay. RPMI with 10 % HS and 5 % FBS was used as cell medium. As negative control 0.1 % Triton X100 was used. For both assays no negative effect on the cell viability could be observed by addition of both compounds.
